# EGFR feedback-inhibition by Ran-binding protein 6 is disrupted in cancer

**DOI:** 10.1038/s41467-017-02185-w

**Published:** 2017-12-11

**Authors:** Barbara Oldrini, Wan-Ying Hsieh, Hediye Erdjument-Bromage, Paolo Codega, Maria Stella Carro, Alvaro Curiel-García, Carl Campos, Maryam Pourmaleki, Christian Grommes, Igor Vivanco, Daniel Rohle, Craig M. Bielski, Barry S. Taylor, Travis J. Hollmann, Marc Rosenblum, Paul Tempst, John Blenis, Massimo Squatrito, Ingo K. Mellinghoff

**Affiliations:** 1 Human Oncology and Pathogenesis Program, Memorial Sloan Kettering Cancer Center, New York, NY 10065 USA; 2 Seve Ballesteros Foundation Brain Tumor Group, Spanish National Cancer Research Centre, 28029 Madrid, Spain; 3 Department of Pharmacology, Weill Cornell Graduate School of Medical Sciences, New York, NY 10065 USA; 4 Molecular Biology Program, Sloan Kettering Institute for Cancer Research, Memorial Sloan Kettering Cancer Center, New York, NY 10065 USA; 5 Department of Neurosurgery, University of Freiburg, 79106 Freiburg, Germany; 6 Department of Neurology, Memorial Sloan Kettering Cancer Center, New York, NY 10065 USA; 7 Division of Cancer Therapeutics, The Institute of Cancer Research, London, SM2 5NG UK; 8 Department of Epidemiology and Biostatistics, Memorial Sloan Kettering Cancer Center, New York, NY 10065 USA; 9 Department of Pathology, Memorial Sloan Kettering Cancer Center, New York, NY 10065 USA; 10 Present Address: Department of Biochemistry and Molecular Biology, Skirball Institute of Biomolecular Biology, New York University School of Medicine, New York, NY 10016 USA; 11 Present Address: Roche Oncology Discovery DTA Molecular Targeted Therapy Group, Basel, 4070 Switzerland

## Abstract

Transport of macromolecules through the nuclear pore by importins and exportins plays a critical role in the spatial regulation of protein activity. How cancer cells co-opt this process to promote tumorigenesis remains unclear. The epidermal growth factor receptor (EGFR) plays a critical role in normal development and in human cancer. Here we describe a mechanism of EGFR regulation through the importin β family member RAN-binding protein 6 (RanBP6), a protein of hitherto unknown functions. We show that RanBP6 silencing impairs nuclear translocation of signal transducer and activator of transcription 3 (STAT3), reduces STAT3 binding to the EGFR promoter, results in transcriptional derepression of EGFR, and increased EGFR pathway output. Focal deletions of the RanBP6 locus on chromosome 9p were found in a subset of glioblastoma (GBM) and silencing of RanBP6 promoted glioma growth in vivo. Our results provide an example of EGFR deregulation in cancer through silencing of components of the nuclear import pathway.

## Abstract (web summary)

The epidermal growth factor receptor (EGFR) signalling is regulated at multiple levels. Here the authors show that the importin RanBP6 acts as a tumor suppressor in Glioblastoma and  regulates EGFR signalling through promoting translocation of STAT3 to the nuclei and repressing EGFR transcription.

## Introduction

The epidermal growth factor receptor (EGFR) is a transmembrane receptor of the ErbB tyrosine kinase family that plays a central role in cell differentiation, proliferation, and survival^1^. EGFR binding to its ligands, e.g., the epidermal growth factor (EGF), leads to phosphorylation and dimerization of the receptor, recruitment of proteins containing Src homology 2 (SH2), and phosphotyrosine-binding (PTB) domains, and activation of multiple downstream signaling pathways, including the mitogen-activated protein kinase (MAPK) pathway, the phosphatidylinositol 3-kinase (PI3K) pathway, and the phospholipase C-γ (PLC-γ) pathway. Activation of EGFR is followed by a series of molecular events that contain EGFR signal strength and duration. These events include endocytosis of the ligand-bound receptor, ubiquitination, and lysosomal degradation of the receptor–ligand complex, and dephosphorylation of the receptor protein by protein tyrosine phosphatases^2^.

Recent studies have challenged the traditional view of EGFR regulation. Structural studies have characterized a distinctive “receptor-mediated” dimerization mechanism and identified allosteric changes that govern the regulation of the intracellular kinase domain^3^. The study of EGFR and its coreceptors at the systems level identified additional EGFR-binding partners, dynamic patterns of pathway activation, and further layers of EGFR regulation through feedback inhibitors and intracellular signal compartmentalization^4–6^. Together, these findings highlight the need for a deeper understanding of EGFR regulation through other signaling pathways.

To identify further mechanisms of EGFR regulation, we characterized the EGFR “interactome” through EGFR immunoaffinity purification and identified Ran-binding protein 6 (RanBP6) as EGFR-associated protein. RanBP6 silencing resulted in increased EGFR RNA and protein levels and augmented EGFR pathway activation in response to EGF. Focal and broad deletions including the *RANBP6* gene locus were identified in glioblastoma (GBM) and RanBP6 silencing accelerated glioma growth in vivo. Taken together, these findings suggest that RanBP6 serves as EGFR regulator that is disrupted in human cancer.

## Results

### RanBP6 interacts with EGFR and Ran-GTPase pathway members

To further advance our understanding of EGFR regulation, we immunoprecipitated endogenous EGFR from whole-cell extracts of A431 human cancer cells, which had been serum starved overnight and then stimulated for 5 min with EGF, and subjected trypsin digests of EGFR-associated proteins to liquid chromatography-tandem mass spectrometry (LC-MS/MS). We identified 431 EGFR-associated proteins in three independent biological replicates. This list of proteins (Supplementary Data 1) comprised the majority of proteins (117/183) detected in a prior examination of the EGFR interactome in A431 cells^7^. About 40% of the proteins (175/431) associating with EGFR were listed as EGFR interactors in the Biological General Repository for Interaction Datasets (BioGRID) and represented well-characterized members of the canonical EGFR pathway, including components of the adaptor protein complex 2 (AP-2), members of the CBL family of E3 ubiquitin-protein ligases, growth factor receptor-bound protein 2 (GRB2), SHC-transforming protein 1 (SHC1), son of sevenless homolog 1 (SOS1), the phosphatidylinositol 4,5-bisphosphate 3-kinase catalytic subunit α and β isoforms (PIK3CA and PIK3CB, respectively), phosphatidylinositol 3-kinase regulatory subunit α (PIK3R1), 1-phosphatidylinositol 4,5-bisphosphate phosphodiesterase gamma-1 (PLCG1), and ERBB receptor feedback inhibitor 1 (ERRFI; also known as mitogen-inducible gene 6 protein).

**Fig. 1:**
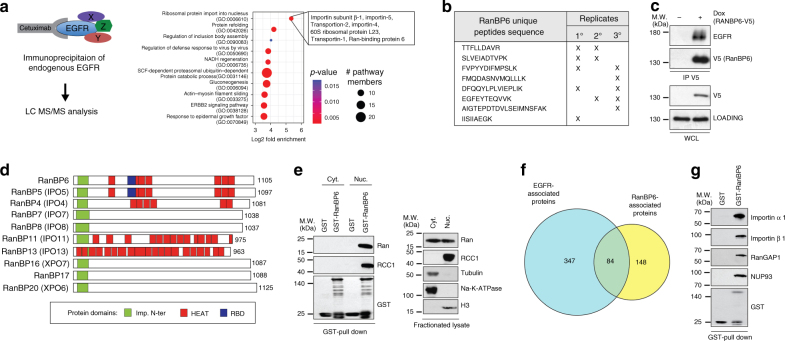
Importin β-like protein RanBP6 interacts with EGFR. **a** Left panel, schematic representation of EGFR immunoaffinity purification and LC-MS/MS analysis in A431. Right panel, plot showing the ten top categories of the gene ontology enrichment analysis of the EGFR-associated proteins. **b** List of the unique peptides for RanBP6 identified in the MS analysis and the replicate in which the peptide was identified are indicated in the table. **c** Co-immunoprecipitation of EGFR and V5 epitope-tagged RanBP6-V5 in A431 cells. Top panel, IP using V5 antibody; bottom panel, immunoblot of whole-cell lysates (WCL). **d** Conserved domains within the family of importin β-related proteins. RanBP6 includes an importin β-like N-terminal domain (Imp. N-ter), seven HEAT repeats, and a putative Ran-binding domain (RBD). The number to the right of each protein shows the total number of amino acids. **e** RanBP6 interacts with nuclear but not cytoplasmic Ran-GTPase. Subcellular fractionation of HEK-293T cells (right panel) shows that Ran is present in both nuclear and cytoplasmic compartments, but only interacts with RanBP6 in the nuclear fraction (left panel). **f** Venn diagram representing overlapping proteins between the RanBP6-V5 and the EGFR immunoaffinity purifications. See Supplementary Data 5. **g** GST pulldown assay confirms the interaction of RanBP6 with importin-α1, importin-β1, RanGAP1, and nuclear pore complex 93 (NUP93) in HEK-293T whole-cell lysates

In addition to these proteins with well-documented roles in EGFR signaling, gene ontology analysis (www.geneontology.org) showed an enrichment of proteins involved in protein import into nucleus (Fig. 1a; Supplementary Data 2). The most highly enriched pathway (GO: 0006610) included importin subunit β-1, importin-5, transportin-2, importin-4, 60s ribosomal protein L23, transportin-1, and RanBP6. Within this group of proteins, only RanBP6 had not previously been reported to bind to EGFR or functionally characterized. We therefore selected it for further study. RanBP6 was identified through eight different unique peptides (Fig. 1b). We cloned a doxycycline (Dox)-inducible RanBP6-V5-tagged complimentary DNA (cDNA) construct and expressed it in A431 cells. Immunoprecipitation with an antibody directed against the V5 epitope confirmed the interaction between RanBP6 and EGFR (Fig. 1c). We also examined the interaction between RanBP6 and EGFR in cells that do not overexpress EGFR. In both (HEK)-293T and LN18 GBM cells, immunoprecipitation of endogenous EGFR pulled down RanBP6 (Supplementary Fig. 1).

RanBP6 contains a putative importin N-terminal domain (Imp. N-ter) (Fig. 1d), suggesting that it is a member of the importin β superfamily^8^. By sequence similarity with RanBP5, which mediates nuclear import of ribosomal proteins^9^, RanBP6 also contains several HEAT (huntingtin, elongation factor 3, the PR65/A subunit of protein phosphatase 2A, and the lipid kinase Tor) repeats and a putative ran-binding domain (RBD). Sequence alignment of the putative RBDs of RanBP6, RanBP5, and importin β1 showed a high sequence homology across different species (Supplementary Fig. 2). We therefore examined interactions of RanBP6 with other members of the Ran-GTPase pathway. Classic nuclear shuttling is mediated by an importin-α∙β complex where importin α recognizes a cargo protein containing a nuclear localization signal and these two proteins then form a ternary complex with importin β1. The ternary complex is dissociated in the nucleus and cargo protein released after binding of importin β1 to nuclear Ran-GTP^10^. Importin-β and β-like importins can mediate nuclear translocation without the assistance of importin-α^11,12^. We therefore examined the association of RanBP6 with several members of the RanGTPase-mediated nuclear transport pathway. Pulldown assays of GST-bound RanBP6 with nuclear and cytoplasmic fractions of HEK-293T cells showed that RanBP6 bound Ran only in the nuclear fraction, where Ran is predominantly GTP-bound. We also observed an interaction between RanBP6 and RCC1, a guanine nucleotide exchange factor that mediates the conversion of RanGDP to RanGTP in the nucleus (Fig. 1e).

To gain a broader view of proteins that interacted with RanBP6, we affinity-purified RanBP6 from A431 cells expressing a Dox-inducible RanBP6-V5 construct and performed LC-MS/MS analysis of four independent experiments. We observed interactions of RanBP6 with 232 proteins, including EGFR (Supplementary Data 3). This list of proteins was highly enriched for gene ontology pathways related to protein targeting to membranes (Supplementary Data 4). A considerable subset of proteins which associated with EGFR in our prior analysis of the EGFR interactome (84/431) also associated with RanBP6 (Fig. 1f; Supplementary Data 5). Interestingly, this list of proteins did not include any of the canonical EGFR pathway members, but did include nuclear pore complex protein Nup93 and multiple components of the SEC61 protein complex (Sec61α1, signal sequence receptor subunit α and δ), which facilitates movement of EGFR between the cytoplasm and the endoplasmic reticulum (ER) in the process of routing the receptor toward the nucleus^13–15^. Using GST-RanBP6 fusion protein as bait, we confirmed in whole-cell lysates the interaction between RanBP6 and nuclear pore complex 93 (Nup93), importin subunit α-1 (importin α1), importin subunit β-1 (importin β1), and the GTPase-activating protein RanGAP1, a GTPase-activating protein, which hydrolyzes RanGTP into RanGDP in the cytoplasm (Fig. 1g). Taken together, our experiments identify RanBP6 as EGFR-interacting protein and member of the Ran-GTPase nuclear transport pathway.

### RanBP6 represses *EGFR* transcription and EGFR signal output

**Fig. 2:**
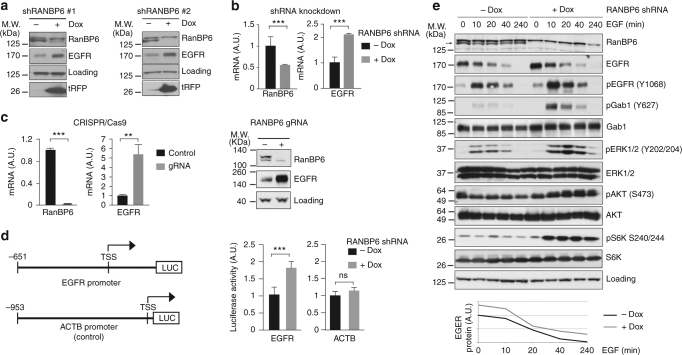
RanBP6 regulates EGFR levels and EGFR signal output. **a** RanBP6 knockdown (KD) raises EGFR protein levels in HEK-293T cells. Dox doxycycline. **b** RanBP6 KD increases *EGFR* mRNA levels in HEK-293T cells. Shown are RT-qPCR results. **c** CRISPR/Cas9-mediated knockout of RanBP6 increases *EGFR* mRNA (left panel) and EGFR protein (right panel) levels in HEK-293T cells. **d** RanBP6 KD increases transcription of a luciferase reporter gene from *EGFR* promoter, but not from the β-actin (*ACTB*) promoter, in HEK-293T. **e** RanBP6 KD increases activation of the EGFR downstream signaling pathways and does not impair EGF-induced EGFR degradation in HEK-293T cells. Upper panel, immunoblot of whole-cell lysates serum starved for 16 h and then stimulated with EGF (100 ng/ml) for the indicated time points; Lower panel, densitometric analysis of EGFR. Data in bar graphs are represented as mean ± SD (*n* ≥ 3). Student’s *t* test: ***p < 0.001; **p< 0.01; ns not significant

Several proteins that bind EGFR, such as CBL family members or ERBB receptor feedback inhibitor 1, play critical roles in EGFR regulation^4–6^. To determine whether RanBP6 might play a role in regulating EGFR levels or function, we generated HEK-293T sublines expressing two different Dox-inducible RanBP6-short hairpin RNAs (shRNAs). RanBP6 knockdown with either hairpin increased EGFR protein levels (Fig. 2a).

We next evaluated the effects of RanBP6 on *EGFR* mRNA levels. Dox-induced knockdown of RanBP6 raised *EGFR* mRNA levels, typically about two-fold (Fig. 2b). Complete RanBP6 depletion using the Clustered Regularly Interspaced Short Palindromic Repeats (CRISPR)/Cas9 system resulted in a more pronounced elevation of EGFR mRNA and protein levels (Fig. 2c). RanBP6 knockdown also increased the expression of a luciferase reporter cloned downstream of the *EGFR* promoter sequence, but had no effect on a control β-actin luciferase reporter (Fig. 2d), suggesting that RanBP6 regulates *EGFR* RNA levels through effects on *EGFR* promoter activity.

Lastly, we examined whether the increase in EGFR levels associated with RanBP6 depletion resulted in increased EGFR pathway output. This was indeed the case, as demonstrated by increased phosphorylation of EGFR, the adapter protein Gab1, and downstream EGFR pathway members ERK1/2, Akt, and S6 kinase following EGF induction (Fig. 2e). Of note, the rate of EGF-induced EGFR protein degradation was comparable in the absence and presence of Dox, further supporting the conclusion that increased EGFR protein levels in RanBP6 knockdown cells were not the result of impaired EGFR protein degradation.

### RanBP6 promotes nuclear translocation of STAT3

**Fig. 3:**
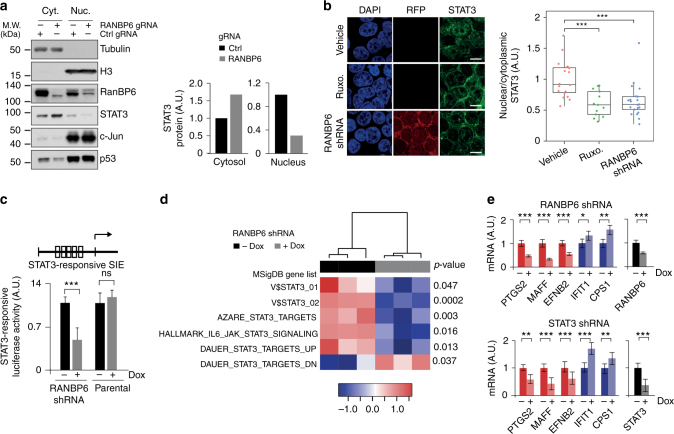
RanBP6 promotes nuclear translocation of STAT3. **a** RanBP6 knockout (KO) increases cytoplasmic STAT3 and lowers nuclear STAT3. Left panel, immunoblots of cytoplasmic (Cyt.) and nuclear (Nuc.) cell fractions; right panel, densitometry of STAT3 immunoblots. **b** RanBP6 KD impairs IL-6-induced nuclear STAT3 translocation. Left panel, confocal immunofluorescence. RFP is used as a reporter for shRNA expression. Right panel, ratios of nuclear/cytoplasmic STAT3 staining (field of views: vehicle, *n* = 18; ruxolitinib, *n* = 13; RANBP6-shRNA, *n* = 21). The janus kinase (JAK) inhibitor ruxolitinib was included as a positive control. Scale bar = 10 μm. **c** RanBP6 KD decreases transcription of STAT3 reporter gene. SIE sis-inducible elements. **d** Gene expression profiling showing the effect of RanBP6 KD on endogenous STAT3 target genes. Heatmap represents the enrichment scores from single-sample gene set enrichment analysis (ssGSEA) of three biological replicates. Student’s *t* test *p*-values (Dox− vs. Dox+) for each gene sets are indicated. **e** Quantitative PCR analysis of the expression of some RanBP6-regulated genes selected from ssGSEA (top panel) confirmed to be regulated by STAT3 (bottom panel). Data are represented as mean ± SD (*n* ≥ 3). Student’s *t* test: ***p < 0.001; **p < 0.01; ns not significant

Members of the β-importin-like protein superfamily transport a variety of cargoes, including transcription factors. We hypothesized that RanBP6 might facilitate the nuclear transport of a transcription factor that regulates *EGFR* promoter activity. We therefore examined the subcellular localization of several transcription factors. We included the transcription factor STAT3 in our analysis because it associated with EGFR in our mass spectrometric analysis (Supplementary Data 1), had previously been shown to associate with EGFR^16–18^, and has been proposed to enter the nucleus through an importin-mediated transport mechanism^19^. RanBP6 knockout cells showed decreased nuclear and increased cytoplasmic STAT3 levels (Fig. 3a). In contrast, we observed no changes in the subcellular localization of several other cancer-related proteins, including the transcription factors p53 and c-Jun, retinoblastoma-associated protein (RB), p27^Kip1^, forkhead box protein O3 (FOXO3), and survivin (Fig. 3a; Supplementary Fig. 3). We also examined the effects of RanBP6 on nuclear translocation of STAT3 by immunofluorescence. RanBP6 knockdown impaired interleukin 6-induced nuclear translocation of STAT3, similar to the ATP-competitive janus kinases (JAK) inhibitor ruxolitinib (Fig. 3b; Supplementary Fig. 4).

We next examined the effect of RanBP6 on STAT3-regulated gene expression. We observed reduced expression of an engineered STAT3 reporter gene following RanBP6 knockdown (Fig. 3c). To evaluate the effects of RanBP6 on the expression of endogenous STAT3 target genes, we used Affymetrix gene expression arrays and single-sample gene set enrichment analysis (ssGSEA). Gene sets that have been reported to be activated by STAT3 (MsigDB, http://www.broadinstitute.org/gsea/msigdb/) showed lower enrichment scores in RanBP6 knockdown cells, whereas gene sets that are negatively regulated by STAT3 (Dauer-STAT3-targets-DN) showed higher enrichment scores in RanBP6 knockdown cells (Fig. 3d). We confirmed these results by quantitative PCR for several of the genes that have been reported to be activated (*PTGS2*, *MAFF*, and *EFNB2*) or repressed (*IFIT1* and *CPS1*) by STAT3. These genes showed similar changes in expression following RanBP6 and STAT3 knockdown, respectively (Fig. 3e).

### RanBP6 represses *EGFR* transcription through STAT3

**Fig. 4:**
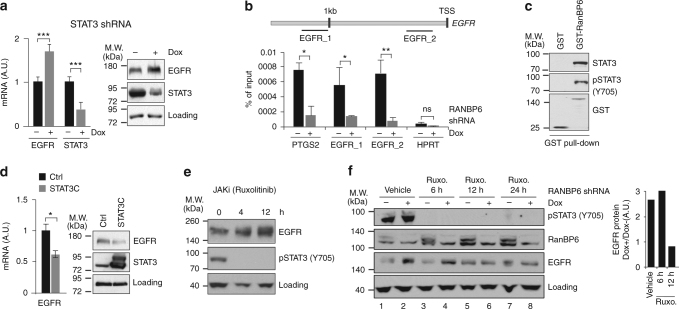
STAT3 represses *EGFR* transcription. **a** STAT3 KD raises *EGFR* mRNA (left panel) and EGFR protein levels (right panel) in HEK-293T. **b** STAT3 binding to the *EGFR* promoter is impaired by RanBP6 KD. Bottom panel, ChIP experiments on the promoter of indicated genes with STAT3 antibody in LN18 cells with Dox-inducible shRanBP6. Plotted values are relative enrichments to % input, measured for two regions (EGFR_1 and EGFR_2) in 1.5 kb upstream of *EGFR* transcriptional start site (TSS) (see top panel). Binding to the *PTGS2* and *HPRT* promoter was used as positive and negative control, respectively. **c** STAT3 and p-STAT3 (Y-705) bind GST-RanBP6 fusion protein in HEK-293T whole-cell lysates. **d** Expression of a constitutive active STAT3 mutant decreases *EGFR* mRNA (left panel) and EGFR protein levels (right panel) in HEK-293T. **e** Inhibition of STAT3 activation by JAK kinase inhibitor ruxolitinib raised EGFR protein level in HEK293T. **f** JAK-STAT blockage with ruxolitinib mitigates the effect of RanBP6 KD on EGFR protein levels in HEK-293T. Right panel, densitometric analysis of EGFR ratio between Dox+ and Dox− samples for each treatment. Data in bar graphs are represented as mean ± SD (*n* ≥ 3). Student’s *t* test: ***p < 0.001; **p < 0.01; ns not significant

Given our findings that RanBP6 regulates nuclear translocation of STAT3 and STAT3-dependent transcription, we wondered whether RanBP6 might mediate transcriptional repression of EGFR through STAT3. We first examined the effect of STAT3 knockdown on *EGFR* mRNA levels using a doxycyline-inducible shRNA construct and observed increased *EGFR* mRNA and protein levels following STAT3 knockdown (Fig. 4a). We next examined whether *EGFR* might be a direct target of transcriptional repression by STAT3. Using the Jaspar transcription profile database (http://jaspar.genereg.net)^20^, we identified multiple putative STAT3-binding sites in a 1.5 kb region upstream to the transcription starting site (TSS) of the *EGFR* gene (Supplementary Table 1). We selected two regions, a proximal and a distal (EGFR_1, −1340:−1111; EGFR_2, −223:−117), for further analysis. By performing an anti-STAT3 ChIP assay, we found that STAT3 protein is recruited to these two specific regions and that the binding is lost upon RanBP6 silencing (Fig. 4b). Similar binding was observed for *PTGS2*, a known STAT3 target gene, but not for the negative control *HPRT*.

Since RanBP6 is associated with both STAT3 and phosphorylated STAT3 (tyrosine 705) (Fig. 4c), we explored whether transcriptional repression of *EGFR* might be mediated by activated STAT3. Expression of a STAT3 mutant with constitutive nuclear localization (STAT3C) was sufficient to lower *EGFR* mRNA levels (Fig. 4d), whereas inhibition of STAT3 phosphorylation with the JAK kinase inhibitor ruxolitinib raised EGFR levels (Fig. 4e). Of note, RanBP6 silencing lost its ability to raise EGFR levels in the setting of sustained (12 h) pharmacological p-STAT3 blockade by ruxolitinib (Fig. 4f, compare EGFR ratios lane 6:lane 5 vs. lane 2:lane 1), suggesting that RanBP6 represses *EGFR* transcription through activated STAT3.

We also explored a potential contribution of exportin-1 (XPO1/CRM1) in this process because XPO1 associated with RanBP6 and EGFR in our mass spectrometric analyses (Supplementary Data 5) and CRM1 inhibition had been reported to reduce STAT3 levels in a breast cancer cell line^21^. However, we observed no effects of CRM1 inhibition on RanBP6 or the levels of STAT3 and acetylated STAT3 (Supplementary Fig. 5).

### EGFR regulation by RanBP6 is disrupted in PTEN-deficient cells

**Fig. 5:**
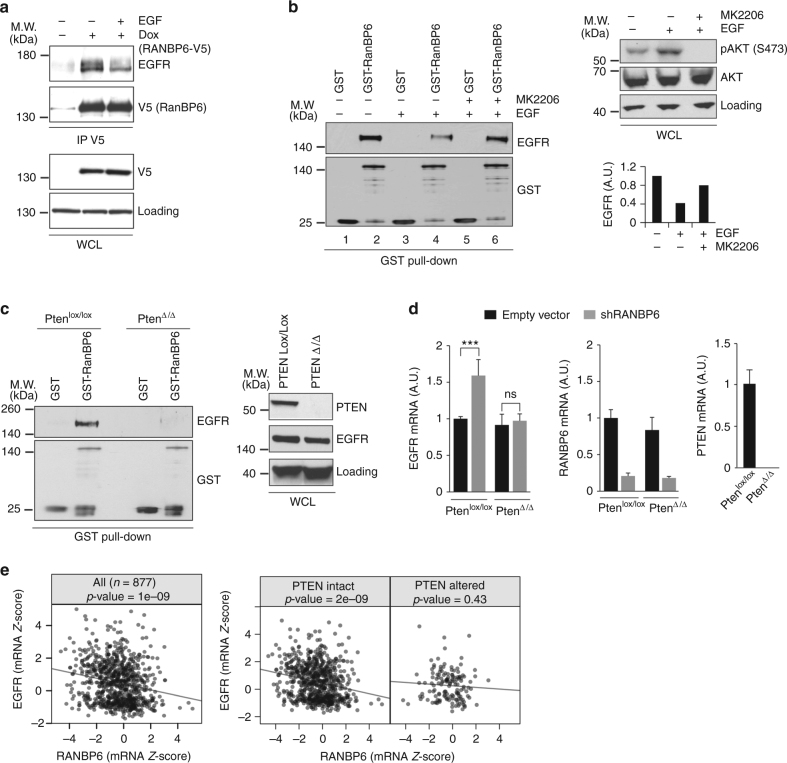
EGFR regulation by RanBP6 is disrupted in PTEN-deficient cells. **a** Co-immunoprecipitation of EGFR and V5 epitope-tagged RanBP6-V5 in A431 cells serum starved and induced with EGF (100 ng/ml) for 5 min. Top panel, IP using V5 antibody; bottom panel, immunoblot of whole-cell lysates (WCL). **b** Interaction of GST-RanBP6 fusion protein with EGFR is Akt dependent. Left panel, GST pulldown; right lower panel, densitometric quantification of GST pulldown; right upper panel, immunoblot of whole-cell lysate. **c** PTEN loss disrupts interaction of GST-RanBP6 fusion protein with EGFR. Left panel, GST-RanBP6 fusion protein interacts with EGFR in *Pten* ^*lox*/*lox*^ but not *Pten* ^Δ/Δ^ MEFs; Right panel, immunoblot of whole-cell lysates. **d** RanBP6 KD raises *Egfr* mRNA level in *Pten* ^*lox*/*lox*^ but not *Pten* ^Δ/Δ^ MEFs. Left panel, *Egfr* mRNA level; middle panel, *Ranbp6* mRNA level; right panel, *Pten* mRNA level. **e** Left panel, negative correlation between *RANBP6* and *EGFR* mRNA *Z*-score in the Cancer Cell Line Encyclopedia (*n* = 877, Pearson product–moment correlation *r* = −0.203, *p*-value = 1e−09). Right panels, cancer cell lines were stratified accordingly to PTEN status. Inverse correlation between *RANBP6* and *EGFR* mRNA levels only in PTEN-intact cancer cell lines (*n* = 734, Pearson product–moment correlation *r* = −0.22, *p*-value = 2e−09) but not PTEN altered cell lines (*n* = 143, Pearson product–moment correlation *r* = −0.066, *p*-value = 0.43). Data in bar graphs are represented as mean ± SD (*n* ≥ 3). Student’s *t* test: ***p < 0.001; ns not significant

In our initial characterization of the interaction between EGFR and RanBP6 in A431 cells, we noted that EGF stimulation (5 min, 100 ng/ml) reduced the interaction between RanBP6 and EGFR (Fig. 5a). This suggested that RanBP6 might be part on an auto-regulatory mechanism where suppression of *EGFR* transcription by RanBP6 is temporarily inactivated following EGFR activation, perhaps to allow restoration of EGFR protein levels following ligand-induced receptor degradation. Similar to our observation in A431 cells, EGF reduced the association between RanBP6 and EGFR in HEK-293T cells. The effect of EGF on the RanBP6–EGFR association could be rescued by pretreatment of cells with the AKT kinase inhibitor MK-2206 (Fig. 5b).

We next examined the effect of the phosphatase and tensin homolog (PTEN), a negative regulator of the PI3K signaling pathway, on the interaction between EGFR and RanBP6. GST-tagged RanBP6 associated with endogeneous EGFR in lysates from mouse embryonic fibroblasts (MEFs) but not  *Pten* knockout MEFs (Fig. 5c). Loss of *Pten* not only impaired the interaction between EGFR and RanBP6, but also abrogated the effects of RanBP6 knockdown on *Egfr *mRNA levels (Fig. 5d), demonstrating that both RanBP6 functions are PTEN-dependent.

Since PTEN is commonly silenced in cancer^22^, we wondered whether PTEN status might affect the relationship between *EGFR* and *RANBP6* RNA levels in human cancer cell lines. We examined this question across a panel of 877 genetically annotated human cancer cell lines included in the publically available Cancer Cell Line Encyclopedia (CCLE)^23^. Consistent with our findings in isogenic models, we observed an inverse relationship between *RANBP6* and *EGFR* mRNA levels (Fig. 5e, left panel). When cell lines were stratified by PTEN status, the inverse correlation between *RANBP6* and *EGFR* mRNA levels was only present in cancer cell lines without PTEN alteration (Pearson product–moment correlation *r* = −0.22, *p*-value = 2e−09) but not in cell lines with PTEN alteration (Pearson product–moment correlation *r* = −0.066, *p*-value = 0.43) (Fig. 5e, right panels) (Supplementary Data 6). Taken together, our findings suggest that EGFR regulation by RanBP6 is disrupted in the setting of acute (e.g., EGF stimulation) or sustained (e.g., PTEN loss) PI3K pathway activation. Our observation that RanBP6 functions are dependent of the activation state of the PI3K pathway is reminiscent of the observation that PI3K pathway activity regulates the function of RanBP3^24^. Unlike RanBP3, however, RanBP6 does not appear to be the recipient of an AKT-regulated phosphorylation signal since EGFR from lysates of PTEN-deficient cells also failed to bind bacterially purified GST-RanBP6 fusion protein, which is not amenable to posttranslational modification.

### RanBP6 shows tumor suppressor-like activity in glioblastoma

**Fig. 6:**
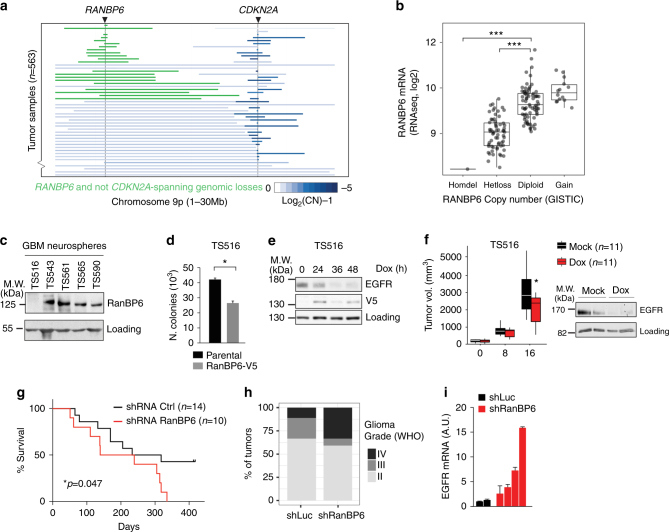
RanBP6 suppresses growth factor output and glioma growth. **a** Focal deletions of the *RANBP6* (left) and *CDKN2A* (right) loci in GBM. **b** Relationship between *RANBP6* copy number and mRNA levels in GBM (*n* = 151); Tukey’s honest significant difference: ***p < 0.001. **c** RanBP6 protein levels in a panel of established patient-derived GBM tumor spheres. The immunoblots of whole-cell lysates are shown. **d** Ectopic expression of RanBP6-V5 in RanBP6-low TS516 GBM neurosphere reduces anchorage-independent growth. **e** Ectopic expression of RanBP6-V5 reduces EGFR protein levels in a time-dependent manner. **f** RanBP6 overexpression reduces tumor growth (left panel) and EGFR expression in a TS516 xenograft model (right panel). Student’s *t* test: *p < 0.05. **g** RanBP6 KD reduces survival in RCAS-tva mouse glioma model. Kaplan–Meier survival curves of PDGFB-induced gliomas generated in Nestin-tva mice injected with either RCAS-RanBP6 shRNA or RCAS-Luciferase shRNA as a control. **h** Tumor grade (WHO classification) of gliomas in the RCAS-tva model. **i** RanBP6 KD increases *Egfr* mRNA in samples from the RCAS-tva mice. Data in bar graphs are represented as mean ± SD (*n *≥ 3)

Aberrant activation of EGFR in human cancer typically occurs through alterations in the *EGFR* gene, but can also be the result of defects in physiologic EGFR feedback regulation^25^. We therefore examined whether RanBP6 exhibits tumor suppressor-like activity. We examined this question in experimental models of GBM because we had observed in several GBMs focal deletions of the *RANBP6* gene locus on chromosome arm 9p (9p24.1). These deletions occurred independently of deletions in *CDKN2A* (Fig. 6a), suggesting that they represented two independent events with selective pressure for the loss of each gene independently. Overall, ~40% of GBMs in the TCGA data sets showed loss of at least one *RANBP6* allele. Copy loss at the *RANBP6* gene locus was most common in the “classical” GBM subgroup (Supplementary Fig. 6), which has been linked to deregulated EGFR activation^26^. Copy loss at the *RANBP6* gene locus was correlated with reduced *RANBP6* mRNA levels (Fig. 6b; Supplementary Data 7). *RANBP6* was lower in tumor tissue compared to non-tumoral brain tissue (Supplementary Fig. 7).

We next examined the relationship between RanBP6 and EGFR expression in GBM. In human GBM tumor sections, we observed an inverse correlation between RanBP6 and EGFR protein levels (Supplementary Fig. 8) and RanBP6 knockdown upregulated EGFR expression in the human GBM cell line LN18 (Supplementary Fig. 9), consistent with earlier results in HEK-293T cells and MEFs.

Western blotting of five patient-derived GBM tumor spheres showed markedly decreased RanBP6 protein levels in one of the five tumor sphere lines (TS516 cells) (Fig. 6c). We stably transduced TS516 cells with a Dox-inducible RanBP6-V5 construct and observed a reduction in soft agar colony formation and reduced EGFR protein levels upon Dox treatment (Fig. 6d, e). Induction of RanBP6-V5 also reduced tumor growth and EGFR expression in subcutaneous TS516 xenografts (Fig. 6f). RanBP6 reconstitution similarly reduced soft agar growth in RanBP6-low SF268 GBM cells (Supplementary Fig. 10).

Lastly, we examined the effect of RanBP6 silencing on in vivo glioma growth using the RCAS-tva mouse glioma model. The RCAS-tva system utilizes an avian leukosis virus-based vectors (RCAS) to mediate gene transfer into cells specifically expressing the tv-a receptor^27^. We injected newborn *N-tva* mice, that express the *Tv-a* under the control of the *Nestin* promoter, a well-known marker of neural stem and progenitor cells, with cells producing the RCAS retroviruses carrying the platelet-derived growth factor-B (PDGFB) in combination with either a mouse RanBP6 shRNA or an shRNA for luciferase as control. RanBP6 knockdown decreased survival, with mice injected with the RanBP6 shRNA living an average of 189 days (*n* = 10) and control mice living 275.5 days (*n* = 14) (*p* = 0.047, log-rank test) (Fig. 6g) and promoted the development of higher-grade gliomas (Fig. 6h; Supplementary Fig. 11). Cells derived from RanBP6 knockdown tumors showed increased *Egfr* mRNA levels (Fig. 6i).

## Discussion

**Fig. 7:**
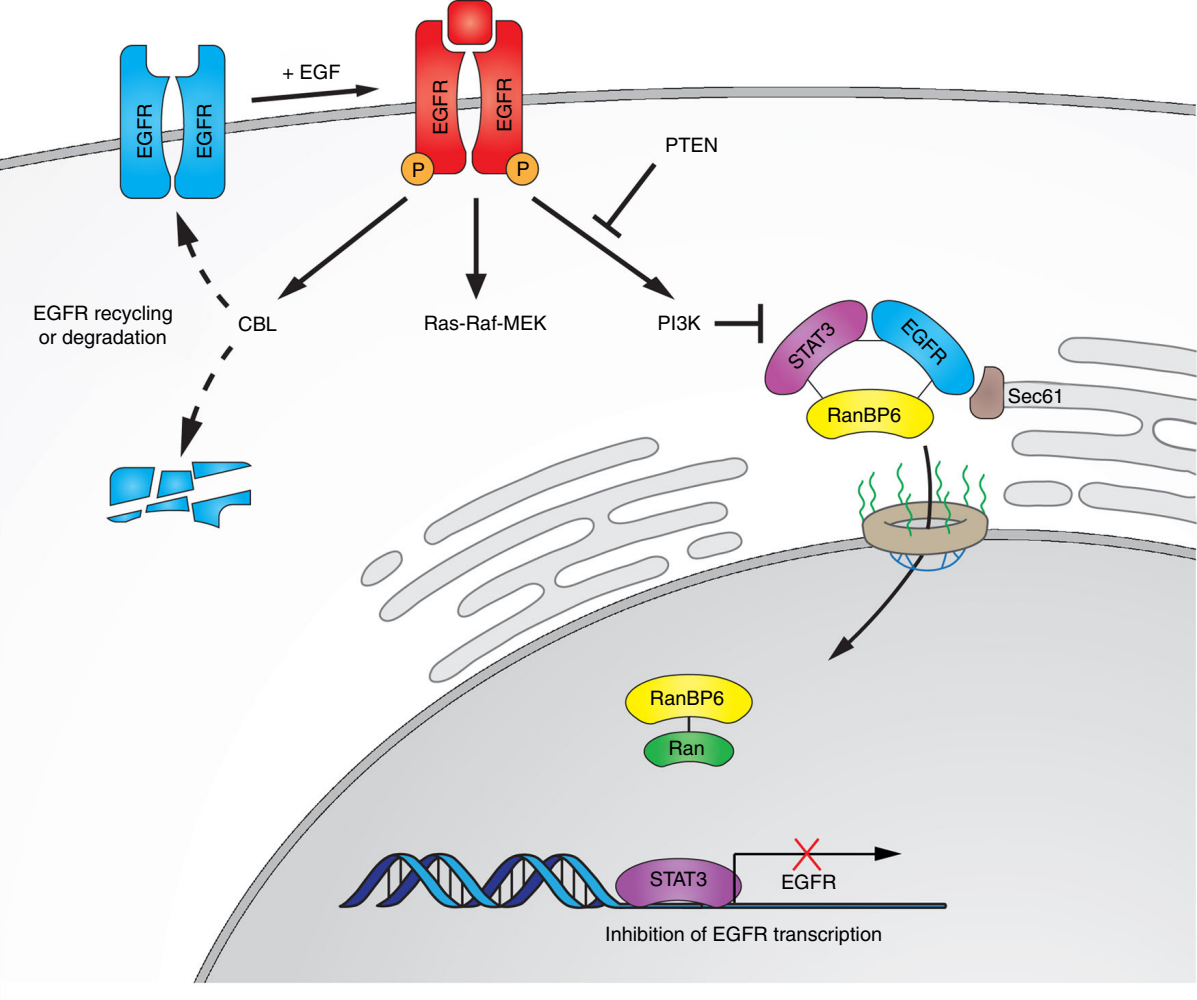
EGFR regulation by RanBP6 (model). A (small) pool of EGF receptors functions as a scaffold for RanBP6-mediated nuclear import of STAT3. Nuclear STAT3 represses EGFR transcription. The solid lines between EGFR–STAT3–RanBP6 and RanBP6–Ran indicate protein–protein interactions (i.e., not necessarily direct molecular interactions). This mechanism of EGFR regulation serves to repress *EGFR* transcription at steady state and is inactivated when the cellular demand for *EGFR* transcription increases (e.g., following EGF-induced receptor protein degradation). Cancer cells inactivate this physiologic mechanism of EGFR regulation through deletion of the *RANBP6* gene or silencing of PTEN (which disrupts the EGFR–RanBP6 interactions)

Our study introduces RanBP6, a protein with currently unknown functions, as member of the importin β superfamily and EGFR regulator. EGFR continuously cycles between the plasma membrane and the endosomal compartment. Activation of EGFR is followed by a series of molecular events that contain EGFR signal strength and duration. In parallel, EGFR signaling is reinforced through the induction of autocrine ligands, which are unable to induce EGFR downregulation and through an increase in EGFR mRNA levels^28–30^. These positive feedback mechanisms aim to restore EGFR levels, work on the same time scale as negative feedback mechanisms, and protect the robustness of ligand-induced mitogenic stimulation^31^. Our results suggest that RanBP6 contributes to cellular EGFR homeostasis by constitutively repressing EGFR transcription and being “switched off” in the setting of increased cellular EGFR demand such as ligand-induced EGFR degradation (Fig. 7).

EGFR has been reported to localize to the nucleus (for review see ref. ^32,33^) through a process that involves trafficking of EGFR from the plasma membrane to the ER, binding to the Sec61 translocon, retro-translocation from the ER to the cytoplasm, and association with importin β^13^. Once in the nucleus, EGFR has been shown to act as a transcriptional co-activator for several genes^17,34^. Our data is consistent with previous findings that EGFR may serve as a scaffold to shuttle a fraction of STAT3 molecules toward the nucleus^16,35–37^ and that STAT3 molecules enters the nucleus through an importin-mediated nuclear transport mechanism^19^. Our observation that STAT3 is a direct transcriptional repressor of EGFR, which has not previously been reported, is consistent with the recent report of increased EGFR signaling following JAK-STAT inhibition^38^ and may have implications for strategies to develop STAT3 inhibitors for cancer therapy. Further studies are needed to determine how RanBP6-facilitated nuclear transport of STAT3 affects the substantial repertoire of STAT3-associated cellular functions and dissect transcriptional programs regulated by STAT3 and phosphorylated STAT3^39–41^.

EGFR is one of the first receptor tyrosine kinases linked to human cancer and represents an important drug target in oncology^42^. Aberrant activation of EGFR and other ErbB receptor family members in cancer is primarily attributed to increased gene copy numbers or gain-of-function mutations in the genes encoding these receptors. However, unbalanced ErbB activation in cancer can also result from defects in EGFR feedback regulation^25^. ERBB receptor feedback inhibitor 1 (ERRFI; also known as mitogen-inducible gene 6 protein), e.g., which encodes a cytosolic protein that directly binds and inhibits ErbB-family receptors, is deleted in cancer and has shown tumor suppressor activity in experimental cancer models^43,44^. Our data suggest that RanBP6 possesses similar tumor suppressor-like activity, at least in GBM. While RanBP6 did not affect a variety of cancer-associated signaling molecules, including other receptor tyrosine kinases (e.g., PDGFRA, PDGFRB, ERBB2, and ERBB3) (Supplementary Fig. 12), we cannot exclude the possibility that the tumor suppressor activity of RanBP6 is mediated by effects that go beyond its effects on EGFR. The nuclear transport machinery is tightly regulated and can be disrupted in cancer through mutations or altered expression of nuclear transport components or disruption of the RanGTP/GDP gradient^45,46^. Taken together, our data identify a link between the Ran-GTPase nuclear transport pathway and key cancer signaling pathways, which warrant further study as inhibitors targeting nuclear transporters enter clinical evaluation as cancer therapeutics^45,46^.

## Methods

### Cell lines and reagents

Epidermoid carcinoma cell line A431, human embryonic kidney HEK-293T, DF1, human glioma cell lines LN18, T98G, A172 were purchased from ATCC. SF268 and SF295 were obtained from NCI. SKMG-3 cells were a gift of Hans Skovgaard (Rigshospitalet, Oslo). HEK-293T and LN18 were authenticated by SNPs analysis. GBM tumor spheres were derived at the MSKCC Brain Tumor Center according to MSKCC IRB guidelines. MEF PTEN^lox/lox^ were kindly provided by Hong Wu (UCLA). All the cell lines were routinely checked for mycoplasma contamination by PCR analysis. DNA fingerprinting was previously performed for authentication of all glioma cell lines^47^. Antibodies to RanBP6 (ab74448; 1:1000), EGFR (ab52894; 1:960 for immunofluorescence staining), RanGAP1 (ab92360; 1:200), and CRM1 (ab24189, 1:1000) were purchased from Abcam. Antibodies to EGFR (#2085; 1:500 for GST-RanBP6 pulldown and 1:1000–5000 for immunoblot), pEGFR Tyr1068 (#3777; 1:1000), Gab1 (#3232; 1:1000), pGab1 Tyr627 (#3231; 1:1000), pErk1/2 Thr202/Tyr204 (#9101; 1:1000), Akt (#9272S; 1:1000), pAkt Ser473 (#4051; 1:1000), S6 (#2317S; 1:1000), pS6 Ser240/244 (#5364; 1:1000), PTEN (#9556; 1:1000), STAT3 (# 12640S; 1:1000), p-STAT3 Tyr705 (#9145S; 1:1000), Ac-STAT3 Lys685 (#2523S; 1:250), H3 (#4499S; 1:5000), p27Kip1 (#2552, 1:500), RB (#9309, 1:1000), and Foxo3a (#2497, 1:1000) were purchased from Cell Signaling. Antibodies to GST (G7781; 1:50,000), V5 agarose affinity gel (A7345; 6 μl antibody/1 mg lysate), importin α (I1784; 1:1000), importin β (I2534; 1:500), Ran (R4777; 1:1000), Vinculin (V9131; 1:10,000), and β-actin (A2228; 1:50,000) were purchased from Sigma. Antibody to V5 (P/N 46-1157; 1:5000) is from Invitrogen. Antibodies against Nup93 (SC-374399; 1:500), RCC1 (SC-55559; 1:1000), STAT3 (SC-482; 1:100 for immunofluorescence staining), tubulin (SC-23948; 1:10,000) and survivin (SC-17779; 1:200) were purchased from Santa Cruz. The AKT inhibitor MK2206, XPO1 inhibitor KPT330 (selinexor), and KPT185 were purchased from Selleckchem, and the JAK inhibitor ruxolitinib (Novartis) was kindly provided by Ross Levine.

### RanBP6 expression vectors and knockdown reagents

To generate the RANBP6 expressing lentiviral construct, RanBP6 was PCR amplified using pBluescriptR-human RanBP6 (Open Biosystems, clone ID 30347107) as template and the primers pLenti6.3-RanBP6-V5 forward and reverse listed in Supplementary Table 2. The amplified product was then transferred into a lentiviral expression plasmid (pLenti6.3/V5-DEST, Invitrogen) with the Gateway recombination technology using the pDONR221 vector as an intermediate vector. Construct was Sanger sequence verified. GST-RanBP6 was generated by sub-cloning the amplified PCR product of human RanBP6 to a digested GST vector (pGEX6.2, GE Healthcare). TRIPZ RanBP6-inducible shRNAs from Open Biosystem (V3THS_374866 and V3THS_374867) were used to knockdown human RanBP6. Mouse-specific RanBP6 hairpin was designed and cloned into the mir30-based retroviral MLP vector (kindly provided by Scott Lowe) and subsequently into the RCAS vector (Supplementary Table 3). RanBP6 cDNA that is resistant to the human hairpin V3THS_374867 was generated by PCR cloning of human RanBP6 cDNA to MSCV-MIGR1-GFP plasmid (Addgene #27490). Three codons inside the hairpin sequence were swapped to generate silence mutations by Site-directed Mutagenesis Kit (Agilent Technologies, catalog #210519-5). TRIPZ STAT3-inducible shRNAs is from Open Biosystem (V3THS_376017).

### Cell line transfections and infections

Adherent lines were grown in DMEM 10% FBS (Omega scientific, FB-11). Neurospheres were grown in NeuroCult NS-A Proliferation Kit (Stem Cell Technology) supplemented with heparin (2 mg/ml), human EGF and bFGF (20 ng/ml each). Lentivirus and retrovirus were generated by co-transfection of retro or lentiviral plasmids and the packaging VSVg for retrovirus and pMD2G and psPAX2 for lentivirus in Gp2-293 using calcium phosphate. High titer virus was collected at 36 and 60 h following transfection and used to infect cells for 12 h. TS516 was spin-infected for 2 h at 1000 rpm. Transduced cells were selected after 48 h from the last infection with blastidicin (2–5 μg/ml), G418 (500–800 μg/ml), and puromycin (3 μg/ml) according to the plasmid antibiotic resistance. DF1 cells were grown at 39 °C in DMEM (ATCC) containing 10% FBS (Sigma, F7524). DF1 cells were transfected with the RCAS viral plasmids, using Fugene 6 Transfection reagent (Roche), accordingly to manufacturer’s protocol. EGF time course experiments were carried out in cells serum starved for 16 h and then stimulated with 100 ng/ml EGF for the indicated time. EGF and Dox were from Sigma.

### Immunoprecipitation and immunoblotting

A431 PTEN isogenic-Dox-inducible RanBP6 V5 cells were induced or not with 1 μg/ml Dox and were lysed 36 h later in JS lysis buffer (50 mM HPES, 150 mM NaCl, 1% glycerol, 1% Triton X-100, 1.5 mM MgCl_2_, 5 mM EGTA). Lysates were precleared by incubation for 1 h at 4 °C with Protein G/A (Calbiochem) blocked in 5% BSA and then incubated with the V5 antibody (Invitrogen) for 2 h, followed by 1 h incubation with Protein G/A. The immunoprecipitates were washed four times with JS lysis buffer and bound proteins were eluted in laemmli buffer. Proteins for immunoblot analysis were run either on 4–12% Bis-Tris SDS–polyacrylamide gel electrophoresis (PAGE) gels (Invitrogen) or on house-made SDS–PAGE gels and transferred to nitrocellulose membrane (Amersham). Membranes were incubated in blocking buffer (5% milk, 0.1% Tween, 10 mM Tris at pH 7.6, 100 mM NaCl) and then with primary antibody either 1 h at room temperature or overnight at 4 °C according to the antibody. Anti-mouse or rabbit-HRP-conjugated antibodies (Jackson Immunoresearch) were used to detect protein by chemiluminescence with ECL (Amersham). Uncropped scan of the main western blots is reported in Supplementary Figs. 14–16.

### GST fusion protein purification and pulldown assay

BL21 cells transformed with pGEX6p2-RanBP6 were grown in 200 ml of LB medium at 37 °C to an *A*_600_ of 0.4–0.7. Protein was induced by culturing in the presence of 1 mM of isopropyl-thio-d-galactopyranoside (IPTG) at 20 °C for 16 h. Bacterial pellets were collected by centrifugation at 7700×*g* for 10 min at 4 °C. Pellets were resuspended in 10 ml of cold lysis buffer (1% Triton X-100, 1 mM of dithiothritol, 1× protease inhibitor cocktail I, and 1× phosphatase inhibitor cocktail in 1× PBS). Resuspended bacterial lysates were sonicated (41% amplitude, 4 pulses of 10 s/cycle) and centrifuged at 12,000 rpm for 15 min at 4 °C. The supernatants were transferred to 15 ml falcon tube and incubated with 50% GST beads slurry at 4 °C for 2–4 h. Supernatants with beads were then sedimented at 500×*g* at 4 °C for 5 min, and washed twice with ice-cold wash buffer, and washed again with 1× PBS without detergent. Beads were finally resuspended in 1–2 bed volumes of GST maintenance buffer (50 mM Tris, 100 mM NaCl 1 mM EDTA, 10% glycerol, 1 mM dithiothritol, and 1× protease and phosphatase inhibitors). The proteins were aliquoted, snap frozen with liquid nitrogen, and stored at −80 °C. About 25 μg of GST empty vector and pGEX-RanBP6 beads were incubated with 500 μg of cell lysates on a rotator in the cold room for 2–4 h. Lysates from HEK-293T were from cells either serum starved for 12 h and stimulated with EGF (100 ng/ml) or grown in full media. The beads were sedimented, washed three times with cold lysis buffer (Cell Signaling, catalog #9803S) with 5 min incubation at 4 °C in between washes, and then lysed with 2× SDS sample buffer (Bio-Rad, #161-0737).

### Mass spectrometry

Lysates from A431 cells serum starved for 24 h and stimulated with EGF (100 ng/ml) for 5 min and from A431-Dox-inducible RanBP6-V5 cell serum starved for 24 h were precleared by ultracentrifugation at 45,000 rpm for 45 min. For the immunopurification of EGFR interactors, 4 mg of lysate was incubated for 2 h and 30 min at 4 °C with 100 μl (slurry 50%) of Cetuximab antibody conjugated to magnetic Dynabeads protein G (Life Technologies). For the immunopurification of the RanBP6 interactors, 2 mg of lysates was incubated overnight with 12 μl anti-V5 agarose affinity gel (Sigma). Supernatants were then removed and beads were washed six times with lysis buffer. The EGFR complexes were eluted in two rounds using 500 mM NH_4_OH and 1 mM EDTA in two rounds of 10 min. The RanBP6–V5 complexes were instead obtained by V5 peptide (Sigma) elution competition in two rounds of 20 min. Negative controls were carried along by precipitating proteins with mouse IgG instead of Cetuximab and with V5 agarose affinity gel in A431 parental cells. Three and four replicates for EGFR and V5 immunoaffinity, respectively, were performed. Elutions were resolved using SDS–PAGE, followed by staining with Coomassie blue and excision of the separated protein bands; in all experiments, prominently stained EGFR band (Mr ~ 170 kDa) and RanBP6-V5 band (Mr ~ 125 kDa) were always excised as an individual protein band for analysis. This was done to enhance the dynamic range encountered during analysis of complex protein mixtures and detection of peptides arising from proteins found in less abundant amounts compared to EGFR and RanBP6-V5. In situ trypsin digestion of polypeptides in each gel slice was performed as described^48^. The tryptic peptides were purified using a 2-µl bed volume of Poros 50 R2 (Applied Biosystems, CA) reversed-phase beads packed in Eppendorf gel-loading tips^49^. The purified peptides were diluted to 0.1% formic acid and then subjected to nano-liquid chromatography coupled to tandem mass spectrometry (nanoLC-MS/MS) analysis as follows. Peptide mixtures (in 20 µl) were loaded onto a trapping guard column (0.3 × 5 mm Acclaim PepMap 100 C18 cartridge from LC Packings, Sunnyvale, CA) using an Eksigent nano MDLC system (Eksigent Technologies, Inc., Dublin, CA) at a flow rate of 20 µl/min. After washing, the flow was reversed through the guard column and the peptides eluted with a 5–45% acetonitrile gradient over 85 min at a flow rate of 200 nl/min, onto and over a 75-micron × 15-cm fused silica capillary PepMap 100 C18 column (LC Packings, Sunnyvale, CA). The eluent was directed to a 75-micron- (with 10-micron orifice) fused silica nano-electrospray needle (New Objective, Woburn, MA). The electrospray ionization needle was set at 1800 V. A linear ion quadrupole trap-Orbitrap hybrid analyzer (LTQ-Orbitrap, ThermoFisher, San Jose, CA) was operated in automatic, data-dependent MS/MS acquisition mode with one MS full scan (450–2000 *m/z*) in the Orbitrap analyzer at 60,000 mass resolution and up to 10 concurrent MS/MS scans in the LTQ for the 10 most intense peaks selected from each survey scan. Survey scans were acquired in profile mode and MS/MS scans were acquired in centroid mode. The collision energy was automatically adjusted in accordance with the experimental mass (*m/z*) value of the precursor ions selected for MS/MS. Minimum ion intensity of 2000 counts was required to trigger an MS/MS spectrum; dynamic exclusion duration was set at 60 s. Initial protein/peptide identifications from the LC-MS/MS data were performed using the Mascot search engine (Matrix Science, version 2.3.02; www.matrixscience.com) with the human segment of Uniprot protein database (20,273 sequences; European Bioinformatics Institute, Swiss Institute of Bioinformatics and Protein Information Resource). The search parameters were as follows: (i) two missed cleavage tryptic sites were allowed; (ii) precursor ion mass tolerance = 10 ppm; (iii) fragment ion mass tolerance = 0.8 Da; and (iv) variable protein modifications were allowed for methionine oxidation, cysteine acrylamide derivatization, and protein N-terminal acetylation. MudPit scoring was typically applied using significance threshold score *p* < 0.01. Decoy database search was always activated and, in general, for merged LS-MS/MS analysis of a gel lane with *p* < 0.01, false discovery rate averaged around 1%. Scaffold (Proteome Software Inc., Portland, OR), version 4_4_4 was used to further validate and cross-tabulate the tandem mass spectrometry- (MS/MS) based peptide and protein identifications. Protein and peptide probability was set at 95% with a minimum peptide requirement of 1.

### Gene ontology analysis

The gene ontology enrichment was performed using the Gene Ontology Consortium website (www.geneontology.org), through the analysis tools from the PANTHER Classification System, by uploading the list of the Uniprot_IDs of the proteins identified in the mass spectrometry experiments. The enrichment results were filtered to reduce the number of redundant GO classes, by using the “Clusterprofiler” and “GOSemSim” packages in R [57,58]. All code used to analyze the data and generate the plots is available at: https://github.com/squatrim/oldrini2017.

### CRISPR/Cas9-mediated knockout of RanBP6

RanBP6 CRISPR constructs were generated with guided RNAs that target human RanBP6 sequence (Supplementary Table 3) and pX330 CRISPR/Cas9 vector (Addgene #42230)^50^. pX330 vector was digested with *Bbs*I and ligated with annealed oligonucleotides. HEK-293T cells were transfected with three different sgRanBP6 constructs. Clonal isolations were performed by serial dilutions (0.5 cells/well). Genomic DNA extractions were performed with the cell lines that are recovered from single cells. Each of the clones was examined by SURVEYOR nuclease assays. The PCR products that were amplified from SURVEYOR primers (Supplementary Table 3) were further validated by Sanger sequence to confirm the indels. Out of all the clones that were generated by three independent sgRNAs, we selected the one that has the best knockout efficiency for further experiments.

### Subcellular fractionation assay

Cytoplasmic and nuclear fractions of HEK-293T cells serum starved for 12 h and treated either with EGF (100 ng/ml) for 15 min or IL-6 (10 ng/ml) for 30 min were prepared with Nuclear Extract Kit (Active Motif, #40010.) The cytoplasmic fractions were extracted with hypotonic buffer. The nuclear pellets were stringently washed four times before addition of nuclear lysis buffer, vortexed, and briefly sonicated (10% amplitude for 5 s) before 30 min incubation on a rotator at 4 °C. For subcellular analysis of STAT3, the lysates were normalized to protein concentration. For GST-RanBP6 pulldown with Ran and RCC1, the fractionated lysates were normalized to the cell number (cytoplasm:nuclear = 50:1).

### Reverse transcription quantitative PCR

RNA was isolated with TRIzol reagent (Invitrogen) according to the manufacturer’s instructions. For reverse transcription PCR (RT-PCR), 500 ng of total RNA was reverse transcribed using the High Capacity cDNA Reverse Transcription Kit (Applied Biosystems). The cDNA was used for quantitative PCR using SYBR Green ER Kit (Invitrogen) according to the manufacturer’s instructions. Quantitative PCRs were run and the melting curves of the amplified products were used to determine the specificity of the amplification. The threshold cycle number for the genes analyzed was normalized to GAPDH and HPRT. Sequences of the primers used are listed in Supplementary Table 4 and primers for human RANBP6 (PPH13358B), ERBB2 (#PPH00209B-200), and ERBB3 (#PPH00463B-200) are from Qiagen.

### Luciferase assay

The promoter constructs of *EGFR* and actin (*ACTB*) were purchased from SwitchGear Genomics (product ID: #S714178 and #S717678). For measuring *EGFR* promoter activity, HEK-293T cells expressing Dox-inducible shRanBP6 were either treated with or without Dox for 72 h, and were further serum starved for 16 h. About 50 ng of actin or *EGFR* promoter construct and 10 ng of cypridina control were co-transfected to the cells with Fugene. The luciferase activities of renilla and cypridina were measured 48 h after transfection by following the manufacturer’s protocol (LightSwitch Dual Assay System, SwitchGear Genomics #DA010). STAT3 reporter for measuring the transcriptional activity of STAT3 was purchased from Qiagen (#CCS-9028L). For STAT3 reporter assay, both HEK293T and HEK293T-RanBP6 cell lines were treated with or without Dox for 72 h. Both of the cell lines were transfected with 100 ng of STAT3 reporter construct. The luciferase assay was developed by using Dual-Glo Luciferase Assay System from Promega (Catalog #E2920). The cells were seeded at a concentration of 15,000 cells/well in the 96-well plate, and were transfected at 60–80% confluence. Each measurement was done in biological triplicates with SpectraMax M5 multi-mode microplate readers (Molecular Devices).

### Gene expression array and ssGSEA

HEK-293T cells expressing Dox-inducible RanBP6 hairpins were either treated with or without Dox for 72 h, and further serum starved for 16 h. Total RNA was extracted with Qiagen RNeasy Mini Kit. The quality of the RNA was evaluated using Agilent BioAnalyzer RNA nano assay, and the high-quality RNA samples were processed for microarray at the Integrated Genomics Operation (IGO) at MSKCC. In summary, 500 ng of the RNA was reverse transcribed to double-stranded cDNA. The cDNA was used as a template for in vitro transcription with biotin-labeled uridine triphosphate at 37 °C for 16 h. The biotin-labeled cDNA was fragmented, and processed to hybridization cocktail to be hybridized to the GeneChip Human Genome U133 Plus 2.0 arrays (Affymetrix) according to the Affymetrix GeneChip protocol. Each sample was done in biological triplicates. Expression array analysis was completed in R (version 3.2.2) using the Bioconductor suite. The “affy” package was used for robust multi-array average normalization followed by quantile normalization. For genes with several probe sets, the median of all probes had been chosen. Data are available online at NCBI GEO, Accession Number GSE76943. ssGSEA has been performed in R using the “gsva” function of the “gsva” package. STAT3-related gene lists were downloaded from the Molecular Signatures Database (MSigDB) at the Broad institute (http://software.broadinstitute.org/gsea/msigdb). All code used to analyze the data and generate the plots is available at: https://github.com/squatrim/oldrini2017.

### Chromatin immunoprecipitation

ChIP was performed as described in Frank et al.^51^ LN18 cells were treated with or without Dox and starved overnight with DMEM without serum. Cells were fixed with 1% formaldehyde for 15 min, stopped with 0.125 M glycine for 5 min, and washed twice with PBS. Cell pellets were sonicated for 6 min at 20% amplification (15 s on followed by 60 s off) followed by 2 min sonication at 40% (15 s on followed by 60 s off) with a Branson 450 Sonifier. Lysates were precleared with Protein A/G beads (Santa Cruz) and incubated at 4 °C overnight with 5 μg of polyclonal antibody specific for STAT3 (sc-482, Santa Cruz), or normal rabbit immunoglobulins (Santa Cruz). DNA was eluted in 100 μl of water and 5 μl was analyzed by qRT-PCR with SYBR Green (Applied Biosystems). The amplification product was expressed as a percentage of the input for each condition. The *HPRT* gene promoter was used as negative control^52^. Primers used to amplify sequences surrounding predicted binding sites were designed using the Primer3 software (http://frodo.wi.mit.edu/cgi-bin/primer3/primer3_www.cgi) based on STAT3-binding site prediction using the Jaspar transcription profile database (http://jaspar.genereg.net)^20^ and the MatInspector software (http://www.genomatix.de).

### Immunofluorescence

HEK-293T cells were seeded at 10,000 cells/well on 12 mm poly-d-lysine and fibronectin-coated rounded coverslip in 24-well plate and cultured in the presence of 2 μg/ml of Dox for 4 days with the last 16–18 h in serum-starved condition. About 1 μM of ruxolitinib was applied to the culture for 4 h and 10 ng/ml IL-6 for 30 min. Cells were fixed in 3.2% PFA in PBS for 20 min, washed three times in PBS, incubated for 20 min in blocking solution (10% donkey or goat serum in 0.1% Triton-X PBS), incubated for 2 h with 1:100 rabbit anti-STAT3 (Santa Cruz, sc-842) in blocking solution, washed three times in PBS, incubated for 1 h with 1:500 anti-rabbit A488 (Invitrogen) in 0.1% Triton-X PBS, washed three times in PBS, and mounted with Vectashield HM-DAPI (Vector Laboratories, H-1500). Cultures were imaged with Leica TCS SP5-II microscope and analyzed using a standardized Metamorph macro. STAT3 signal was first threshold to select the signal over the background, then the DAPI image was used to subdivide the threshold STAT3 signal into nuclear and cytoplasmic, and ratio was calculated. For the staining of human GBM tissue sections, tumors were formalin-fixed, paraffin-embedded, cut into 5 μm sections, and stained with DAPI (Molecular Probes, D3571), RanBP6 (polyclonal, ab74448, Abcam; 1:200), and EGFR (clone EP38Y, ab52894, Abcam; 1:960). Several fields of view were selected by a neuropathologist for further analysis. Specificity of RanBP6 staining and lack of cross-reactivity for RanBP5 was determined in normal tissue sections and HEK-293T cells transfected with cDNA for RanBP6 or RanBP5. Image acquisition, registration, segmentation, and quantification were performed using the method previously described^53^.

### Soft agar assay

TS516 cells were seeded in triplicates at 300,000 cells/well in Neurocult media containing 0.4% Noble agar (SIGMA A5431) and growth factor supplements (20 ng/ml EGF, 10 ng/ml bFGF) and SF268 at 50,000 cells/well in DMEM 10% FBS. Cells were plated between two layers of Neurocult media and growth factors or DMEM and FBS containing 0.65% Nobel agar. Noble agar layers were containing Dox at 1.2 μg/ml. Colonies were stained 3/4 weeks after plating with either crystal violet (0.005%) (Sigma V5265) and quantified using imagine software (Oxford Optronix) and an image processing algorithm (Charm algorithm, Oxford Optronix).

### Evaluation of glioma growth in vivo

For the TS516 xenograft model, 4–6 weeks old female SCID mice were injected subcutaneously with 10^6^ glioma cells, which were suspended in 100 μl of a 50:50 mixture of growth media and Matrigel (BD #356237). Mice were then randomly assigned to treatment groups (Dox or control). Ntv-a mice, and procedures for RCAS-mediated gliomagenesis have been described previously^54^. Ntv-a pups were injected with a total of 200,000 DF1 cells transfected with various constructs: 100,000 RCAS-PDGFB plus 100,000 RCAS-shRanBP6 or RCAS-shLuc. After injection of the DF1 cells during the newborn period, mice were aged until they developed symptoms of disease (lethargy, poor grooming, weight loss, macrocephaly). Samples in panel i of Fig. 6 are derived from tumors generated in a Ntv-a; Ink4a/Arf null background. RCAS-shRanBP6 and RCAS-shLuc constructs express a EGFP reporter that allowed to isolate the tumor cells by FACS. For the derivation of primary cells for FACS analysis, tumors were digested to a single-cell suspension by 10 min of incubation at 37 °C with 5 ml of papain digestion solution (0.94 mg/ml papain (Worthington), 0.48 mM EDTA, 0.18 mg/ml *N*-acetyl-l-cysteine (Sigma) and 0.06 mg/ml DNase I (Sigma) diluted in Earl’s Balanced Salt Solution (EBSS). After digestion, the enzyme was inactivated by the addition of 2 ml of 0.71 mg/ml ovomucoid (Worthington). The cell suspension was then passed through a 40-μm mesh filter to remove undigested tissue and centrifuged at a low speed (750 r.p.m.) to remove debris and obtain the cell pellet^54^. Cells were then resuspended in 500 μl of PBS to be sorted. All animal experiments were performed according to protocols approved by the Institutional Animal Care and Use Committee of Memorial Sloan Kettering Cancer Center and CNIO-ISCIII Ethics Committee for Research and Animal Welfare (CEIyBA) and they were performed in accordance with the guidelines stated in the International Guiding Principles for Biomedical Research Involving Animals, developed by the Council for International Organizations of Medical Sciences (CIOMS).

### Statistical analysis

Data are presented throughout as mean and SD, except otherwise indicated. Results were analyzed by unpaired two-tailed Student’s *t* tests unless otherwise noted and were considered statistically significant if *p* < 0.05. Kaplan–Meier survival curve was produced with GraphPad Prism; *p*-value was generated using the log-rank statistic.

### Data availability

The microarray data have been deposited in the NCBI GEO database under the accession code GSE76943. The proteomic data have been deposited in the UCSD MassIVE database (https://massive.ucsd.edu/ProteoSAFe/static/massive.jsp) under MassIVE accession IDs MSV000081631 and MSV000081632. The CCLE data referenced during the study are available in a public repository from the cBio Portal^55^ using the “cgdsr” package. The TCGA GBM and REMBRANDT data referenced during the study are available in a public repository from GlioVis data portal (http://gliovis.bioinfo.cnio.es)^56^. All code used to analyze the data and generate the plots is available at: https://github.com/squatrim/oldrini2017. All the other data supporting the findings of this study are available within the article and its Supplementary Information files, and from the corresponding authors upon reasonable request.

## Electronic supplementary material

Description of Additional Supplementary Files

Supplementary Information

Supplementary Data 1

Supplementary Data 2

Supplementary Data 3

Supplementary Data 4

Supplementary Data 5

Supplementary Data 6

Supplementary Data 7
