# Supplementary material for: EGFR feedback-inhibition by Ran-binding protein 6 is disrupted in cancer
Source: Nat Commun. 2017 Dec 11;8:2035. doi: 10.1038/s41467-017-02185-w (PMC5725448; doi:10.1038/s41467-017-02185-w)
Supplement: Supplementary file 1 — Description of Additional Supplementary Files [file 41467_2017_2185_MOESM1_ESM.pdf]

Description of Additional Supplementary Files

File Name: Supplementary Data 1
Description: EGFR Mass spectrometry data

File Name: Supplementary Data 2
Description: Gene Ontology EGFR interacting proteins.

File Name: Supplementary Data 3
Description: RanBP6 interacting proteins.

File Name: Supplementary Data 4
Description: Gene Ontology RanBP6 interacting proteins.

File Name: Supplementary Data 5
Description: Shared EGFR and RanBP6 interacting proteins.

File Name: Supplementary Data 6
Description: Cancer Cell line Encyclopedia (CCLE) data.

File Name: Supplementary Data 7
Description: GBM expression datasets.
