# Supplementary material for: EGFR feedback-inhibition by Ran-binding protein 6 is disrupted in cancer
Source: Nat Commun. 2017 Dec 11;8:2035. doi: 10.1038/s41467-017-02185-w (PMC5725448; doi:10.1038/s41467-017-02185-w)
Supplement: Supplementary file 2 — Supplementary Information [file 41467_2017_2185_MOESM2_ESM.pdf]

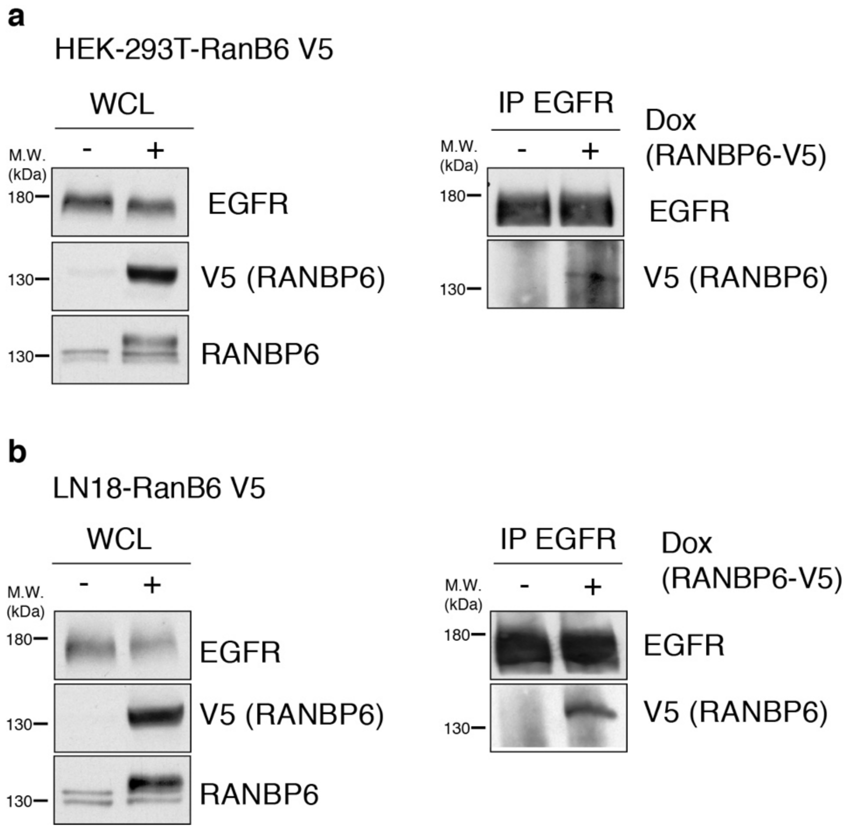

Supplementary Figure 1. RanBP6 association with EGFR. (a) and (b) Co-immunoprecipitation of EGFR and V5 epitope tagged RanBP6-V5 in HEK-293T and LN18 cells, respectively. WCL = whole cell lysates.

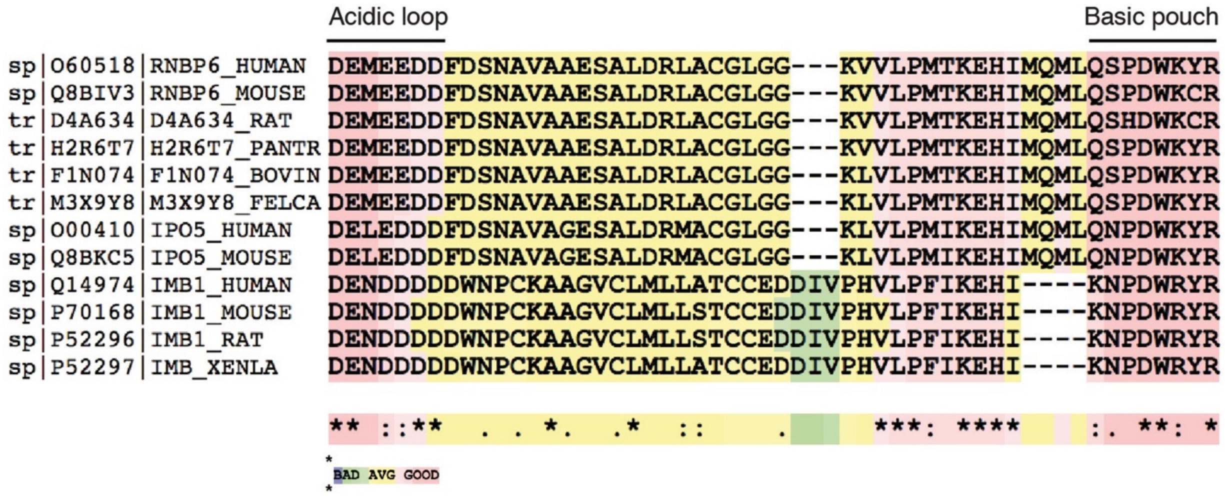

Supplementary Figure 2. Sequence alignment of the Ran Binding Domain (RBD) of RanBP6. Note the high sequence homology between the Ran Binding Domains (RBD) of RanBP5, RanBP6 and importin β1 from different species.

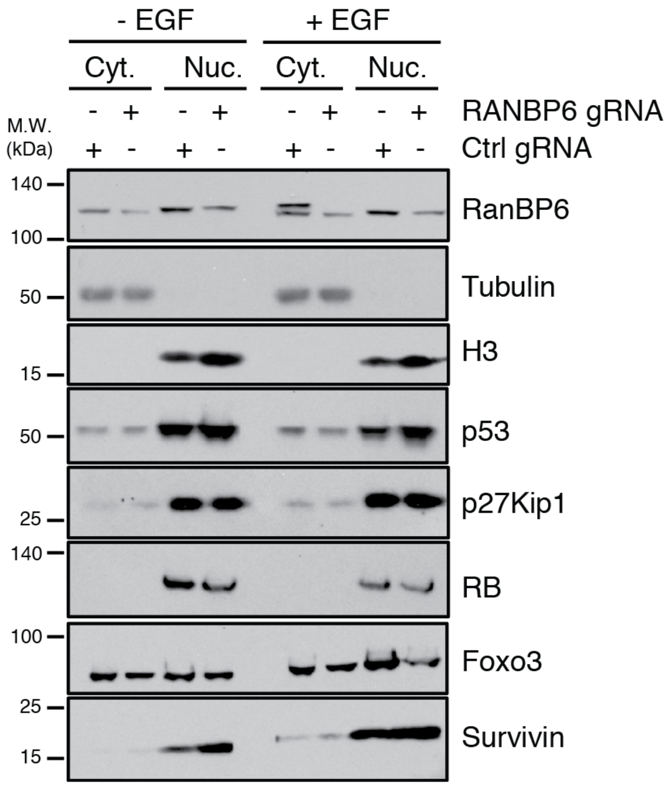

Supplementary Figure 3. RanBP6 depletion does not change nuclear localization of p53, p27^{Kip1}, RB, Foxo3 and Survivin. Shown are immunoblots of subcellular fractionations in HEK293T cells with either control gRNA or RanBP6 gRNA. Cytoplasmic (Cyt.) and nuclear (Nuc.) fractions were immunoblotted with the indicated antibodies. Note that addition of EGF (100 ng/ml, 5 minutes) does not change nuclear localization of these proteins.

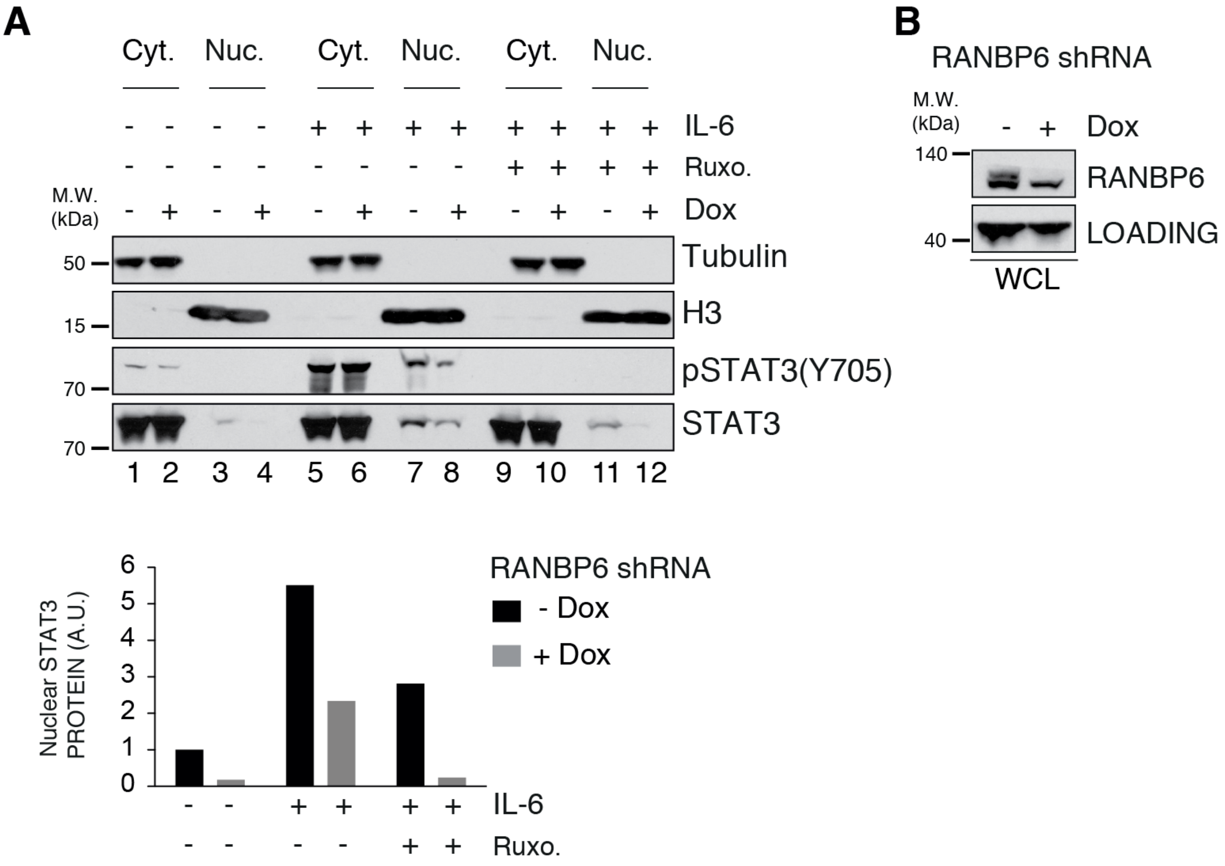

Supplementary Figure 4. RanBP6 knockdown impairs IL-6 induced nuclear translocation of STAT3. (a) Shown are immunoblots *(upper panel)* and densitometric quantification *(lower panel)* of fractionated cell lysates from HEK-293T cells treated with the indicated reagents. Note that the IL-6 induced nuclear localization of STAT3 and p-STAT3 is reduced by pretreatment of cells with the JAK kinase inhibitor ruxolitinib (lane 11 *versus* lane 7). A similar effect is observed following RanBP6 knockdown (lane 8 *versus* lane 7). (b) immunoblot confirms Dox-induced RanBP6 knockdown in the whole cell lysate. Cyt.=cytoplasm. Nuc.=nucleus.

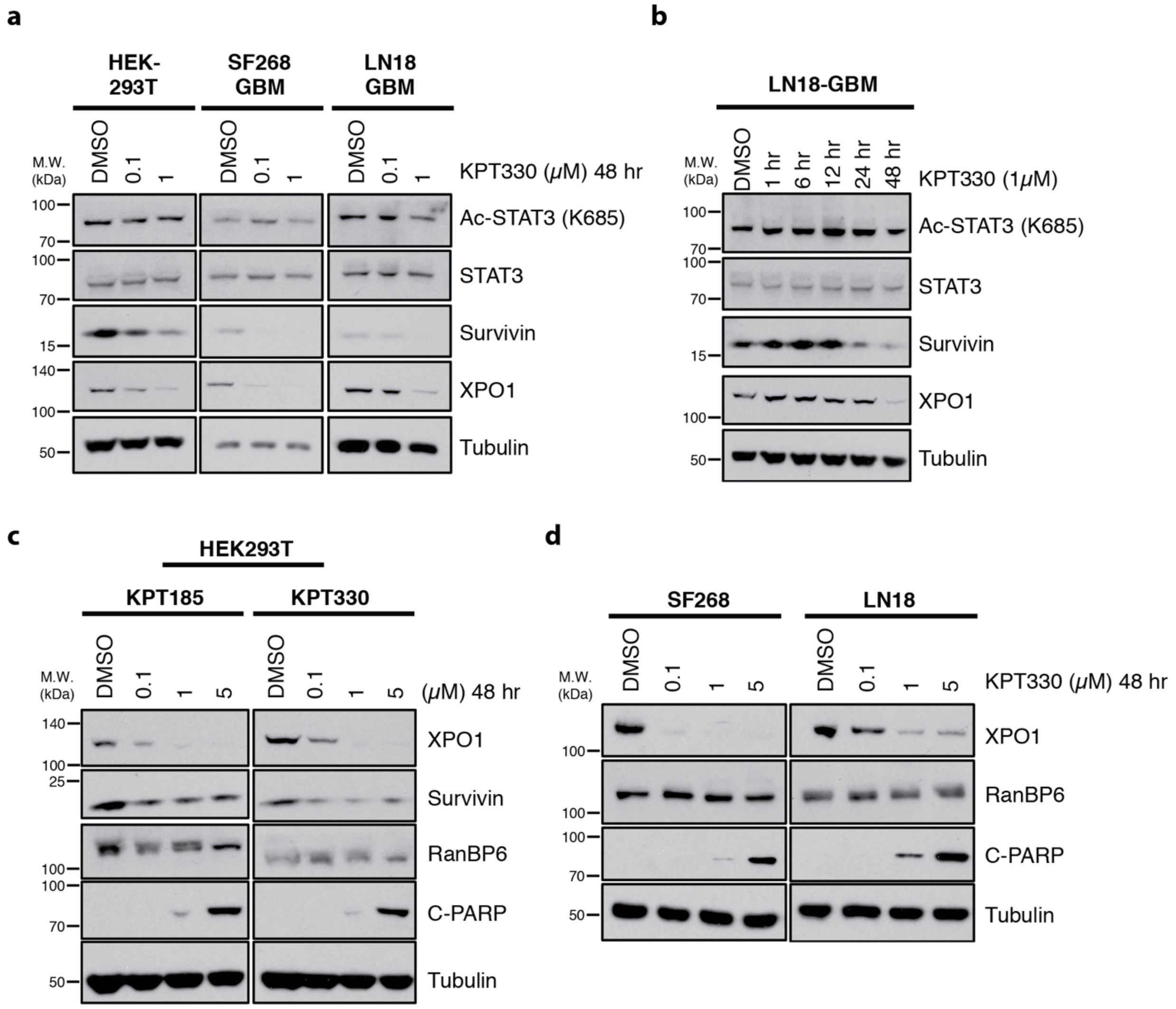

Supplementary Figure 5. XPO1 inhibition does not reduce protein levels of total STAT3 or acetylated STAT3 or RanBP6. (a) Immunoblots of whole cell lystates from the indicated cell lines treated for 48 hours with DMSO or the XPO1 inhibitor KPT-330 (selinexor). (b) Immunoblots of whole cell lysates from LN18 cell line treated for with KPT-330 (1 μM) and lysed at the indicated time points. (c) Immunoblots of whole cell lysates from HEK293T cells treated for 48 hours with the indicated concentrations of two second generation selective inhibitors of nuclear export (SINE)-KPT185 (left) and KPT330 (right). (d) Immunoblots of whole cell lysates from SF268 and LN18 GBM cell lines incubated for 48 hours with the indicated concentrations of KPT330.

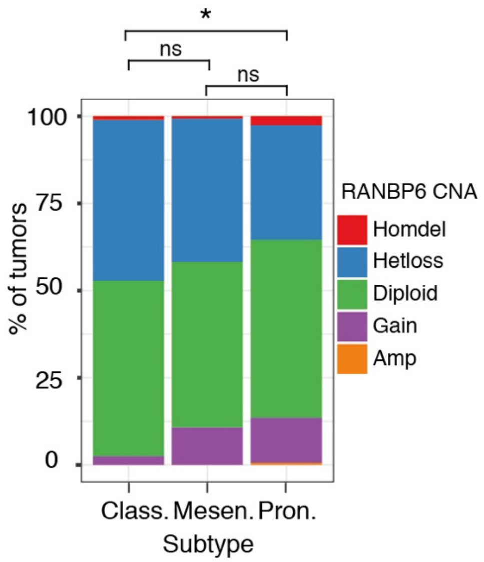

Supplementary Figure 6. *RANBP6* gene copy number alterations in glioblastoma subgroups. *RANBP6* copy number alterations in TCGA samples stratified by GBM subtype (Classical, Mesenchymal and Proneural); ns = not significant, **P* < 0.05, Fisher's exact test (Homdel + hetloss versus all other).

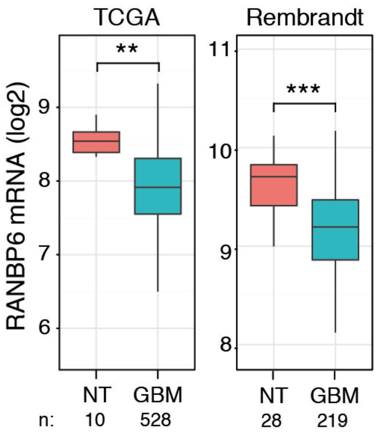

Supplementary Figure 7. RanBP6 RNA levels in glioblastoma compared to normal brain. RANBP6 is down regulated in GBM versus non-tumoral brain control in the TCGA and Rembrandt datasets. Tukey's Honest Significant Difference, ***$P < 0.001$; **$P < 0.01$.

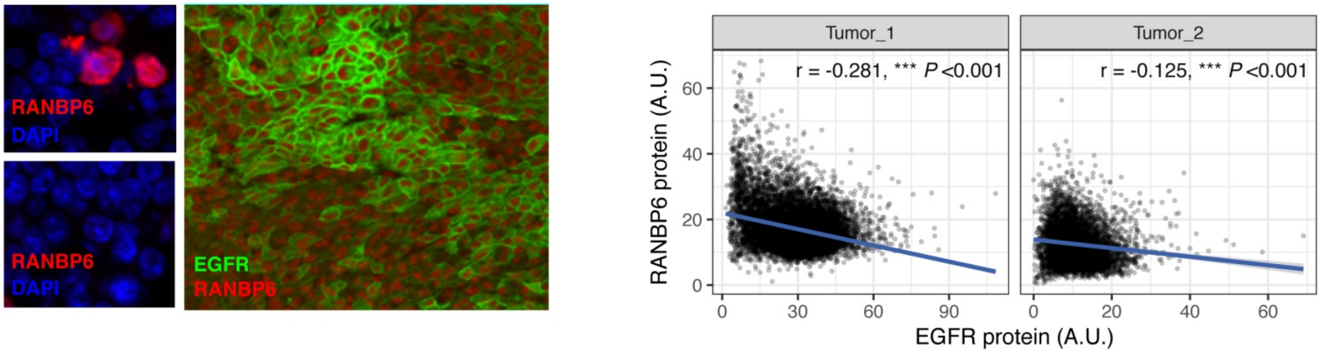

Supplementary Figure 8. Inverse correlation between RanBP6 and EGFR protein correlation in human glioblastoma tissue sections. *Left top panel*: representative immunofluorescence (IF) images of HEK293T cells transfected with RanBP6 cDNA. *Left bottom panel:* representative IF images of HEK293T cells transfected with RanBP5 cDNA. *Middle panel:* representative images of GBM tissue section stained with both EGFR and RanBP6 antibodies. *Right panel:* quantification of EGFR and RanBP6 coexpression at the single-cell level in two human GBMs (Tumor 1, left; Tumor 2, right). Graphed is the staining intensity for EGFR (x-axis) and RanBP6 (y-axis). Each data points represents a single cell. Cells were pooled from multiple regions of interest; r = Pearson product-moment correlation. A.U. = arbitrary units.

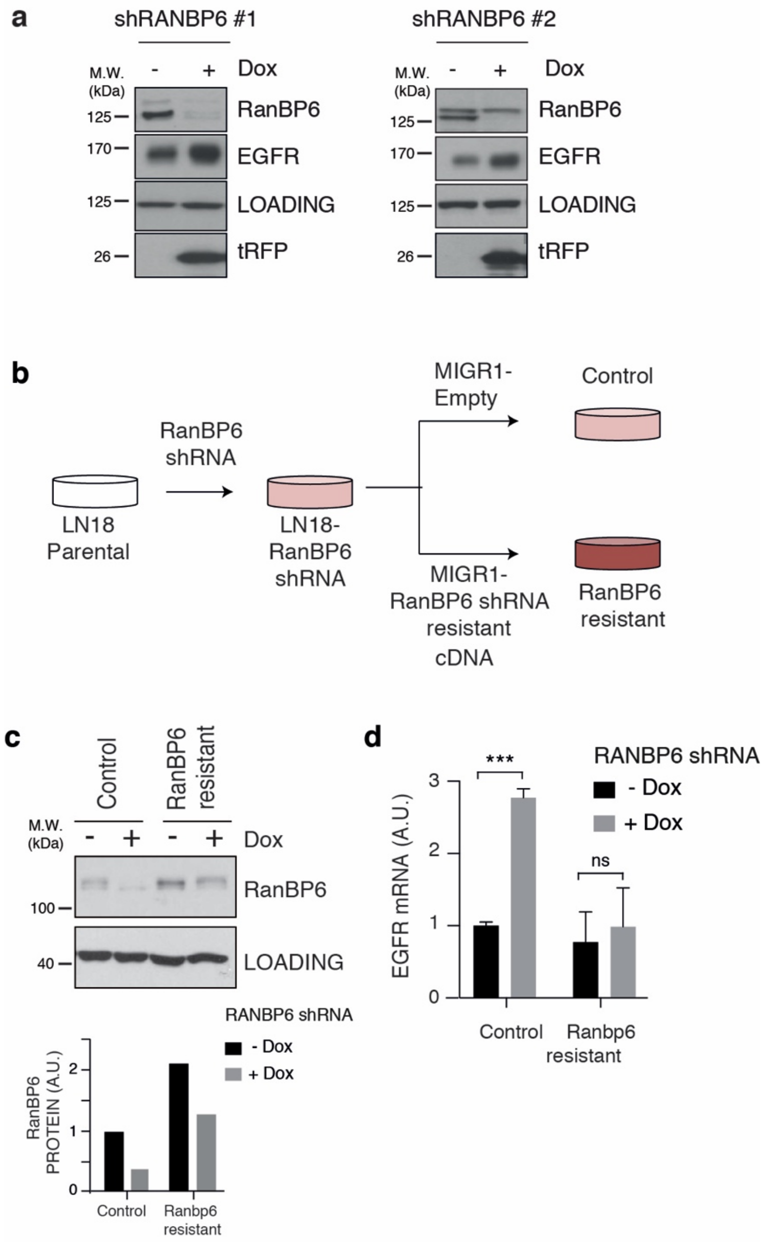

Supplementary Figure 9. RanBP6 represses EGFR transcription in LN18 cells. (a) Depletion of RanBP6 increases EGFR protein levels in LN18 cells expressing two different Dox-inducible RanBP6-shRNAs. (b) Cartoon showing the generation of RanBP6 shRNA resistant cDNA in LN18 cells. (c-d) RanBP6 knockdown increases EGFR transcript levels in LN18 cells and restoration of RanBP6 expression through shRNA-resistant cDNA re-establishes normal EGFR mRNA levels. Data in bar graphs are represented as mean ± SD (n=3). Student's *t* test, ***$P < 0.001$; ns, not significant.

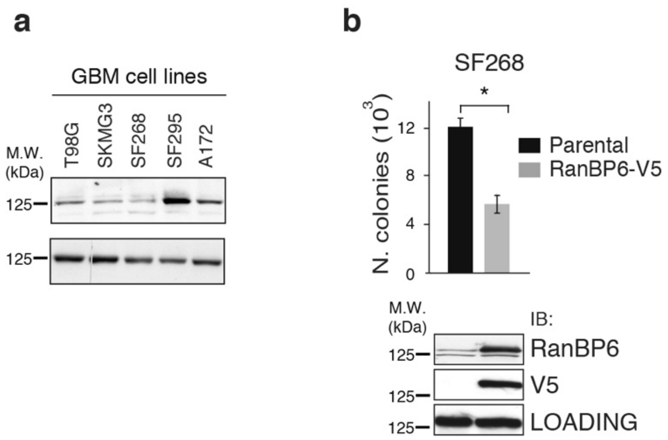

Supplementary Figure 10. RANBP6 restoration retards growth of SF268 GBM cells. (a) Immunoblot showing RanBP6 protein expression level in a panel of GBM adherent cell line. (b) Ectopic expression of RanBP6-V5 in RanBP6-low SF268 GBM cells reduces anchorage-independent growth and lowers EGFR protein levels. Immunoblots from whole cell lysates are shown below the bar graphs. Data in bar graphs are represented as mean ± SD (n=3). Student's *t* test, *$P < 0.05$.

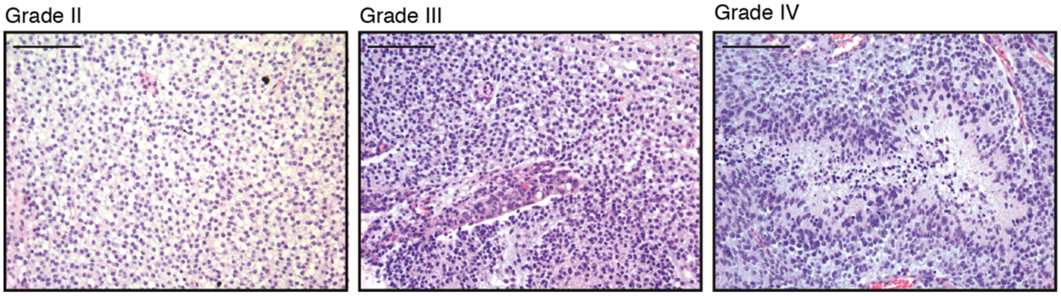

Supplementary Figure 11. RANBP6 silencing increases GBM frequency. Representative H&E images of tumors from the RCAS-PDGF injected mice. Tumors were classified using the World Health Organization (WHO) grading system. Glioblastoma (GBM) are Grade IV.

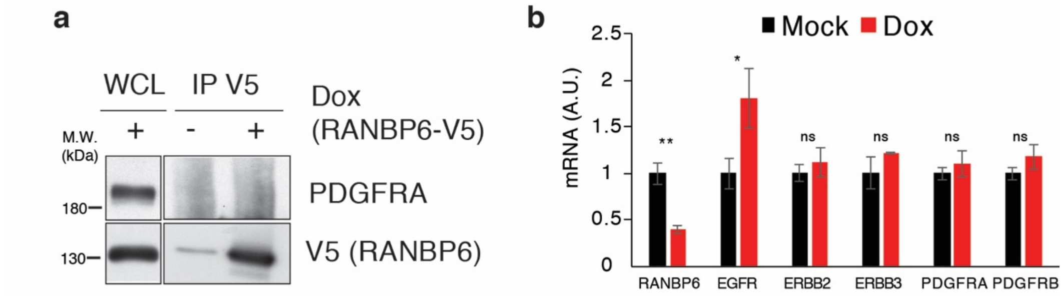

Supplementary Figure 12. RanBP6 and Platelet-derived Growth Factor Receptor (PDGFR). (a) RanBP6 does not associated with PDGFRA. Dox-inducible RanBP6-V5 expression vector was transduced into LN18 cells. RanBP6 was immunoprecipitated using a V5 antibody and protein complexes were the immunoblotted with a PDGFRA and V5 antibody. WCL = whole cell lysates. (b) RanBP6 knockdown does not increase PDGFRA or PDGFRB transcription. EGFR, ERBB2, and ERBB3 are included as controls. Shown are results in LN18 glioblastoma cells. Data in bar graphs are represented as mean ± SD (n=3). Student's *t* test (Dox- versus Dox +), $^{**}P < 0.01$; $^{*}P < 0.05$; ns, not significant.

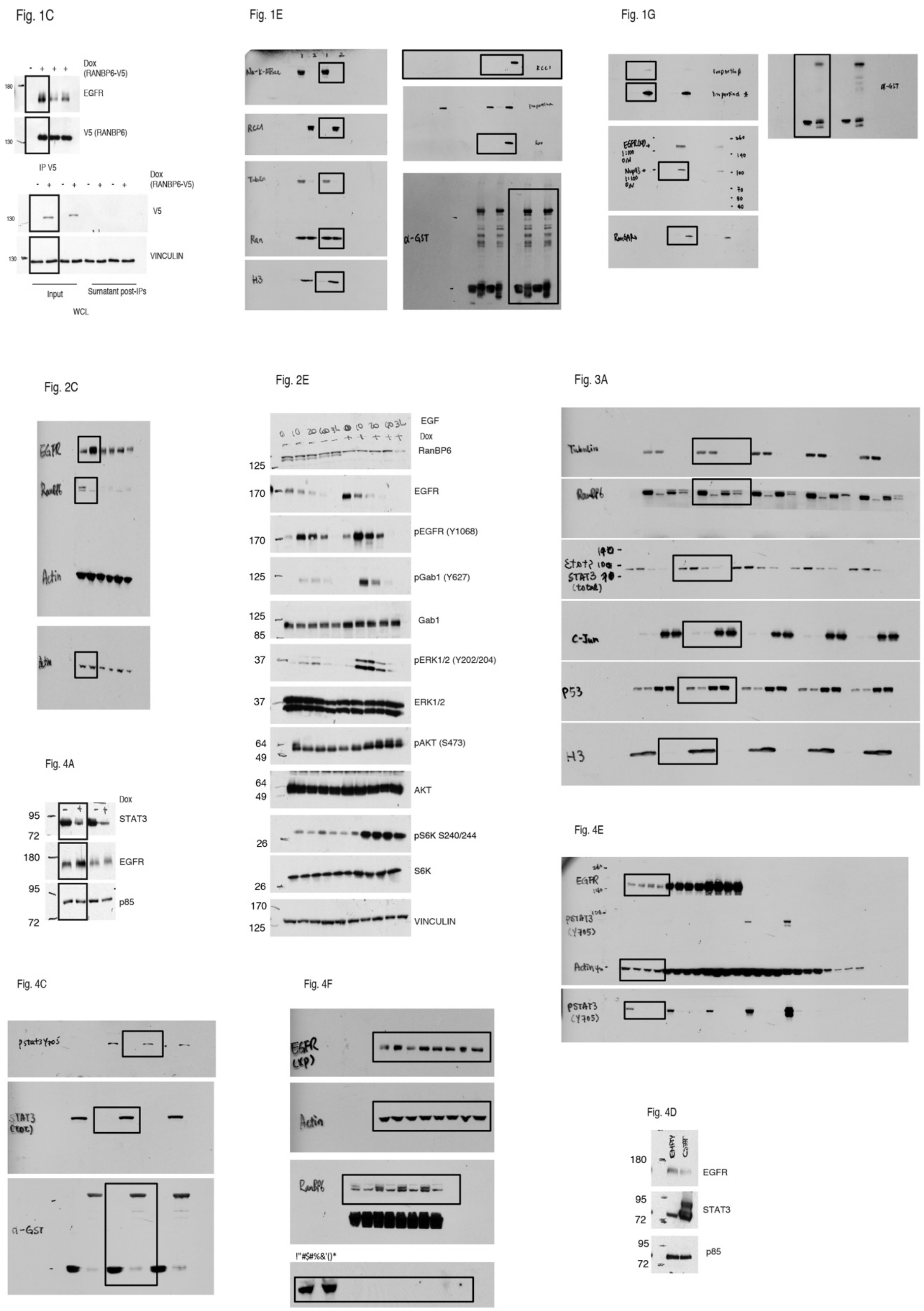

Supplementary Figure 13. Uncropped western blot images for Figures 1 to 4

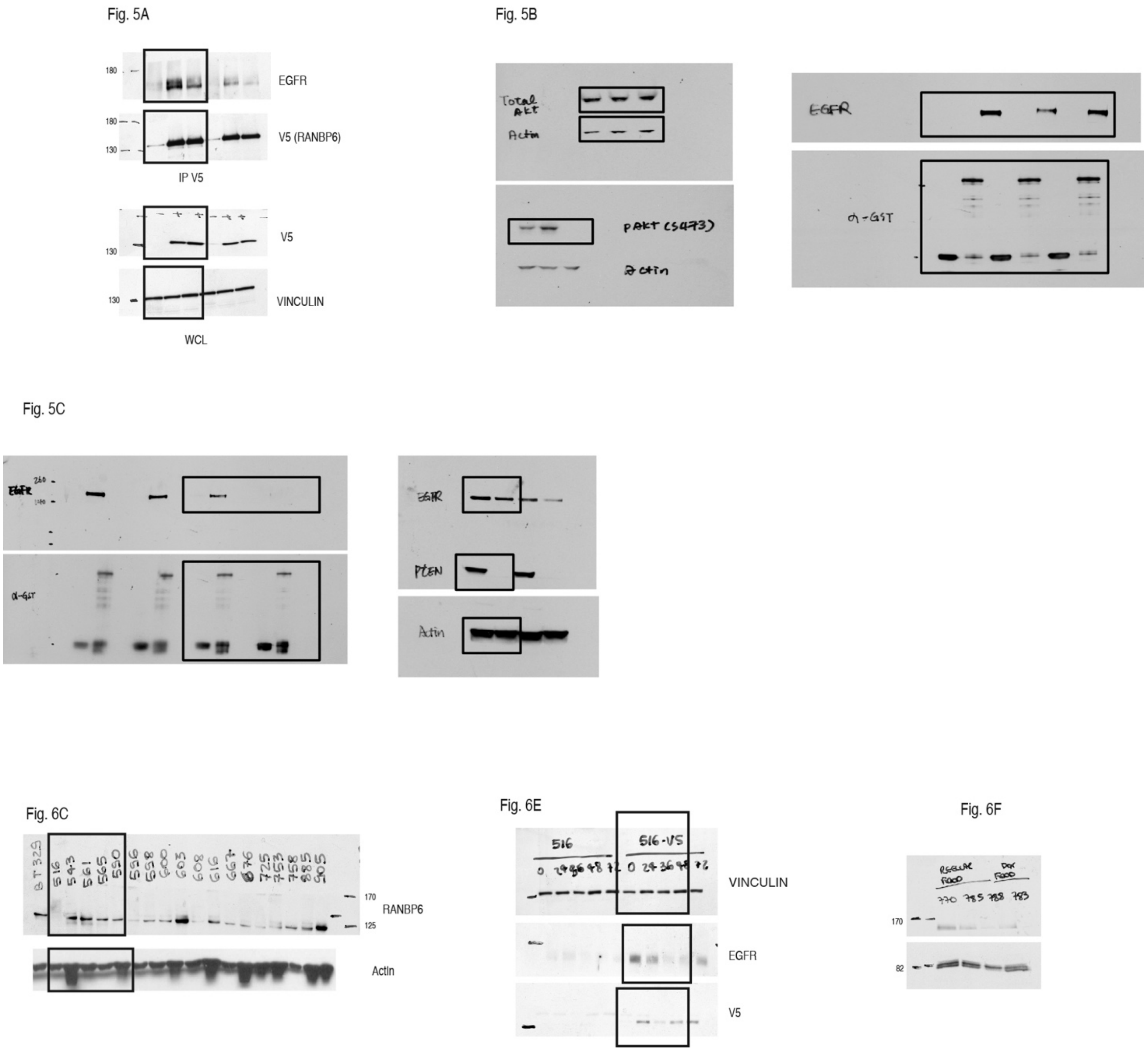

Supplementary Figure 14. Uncropped western blot images for Figures 5 to 6

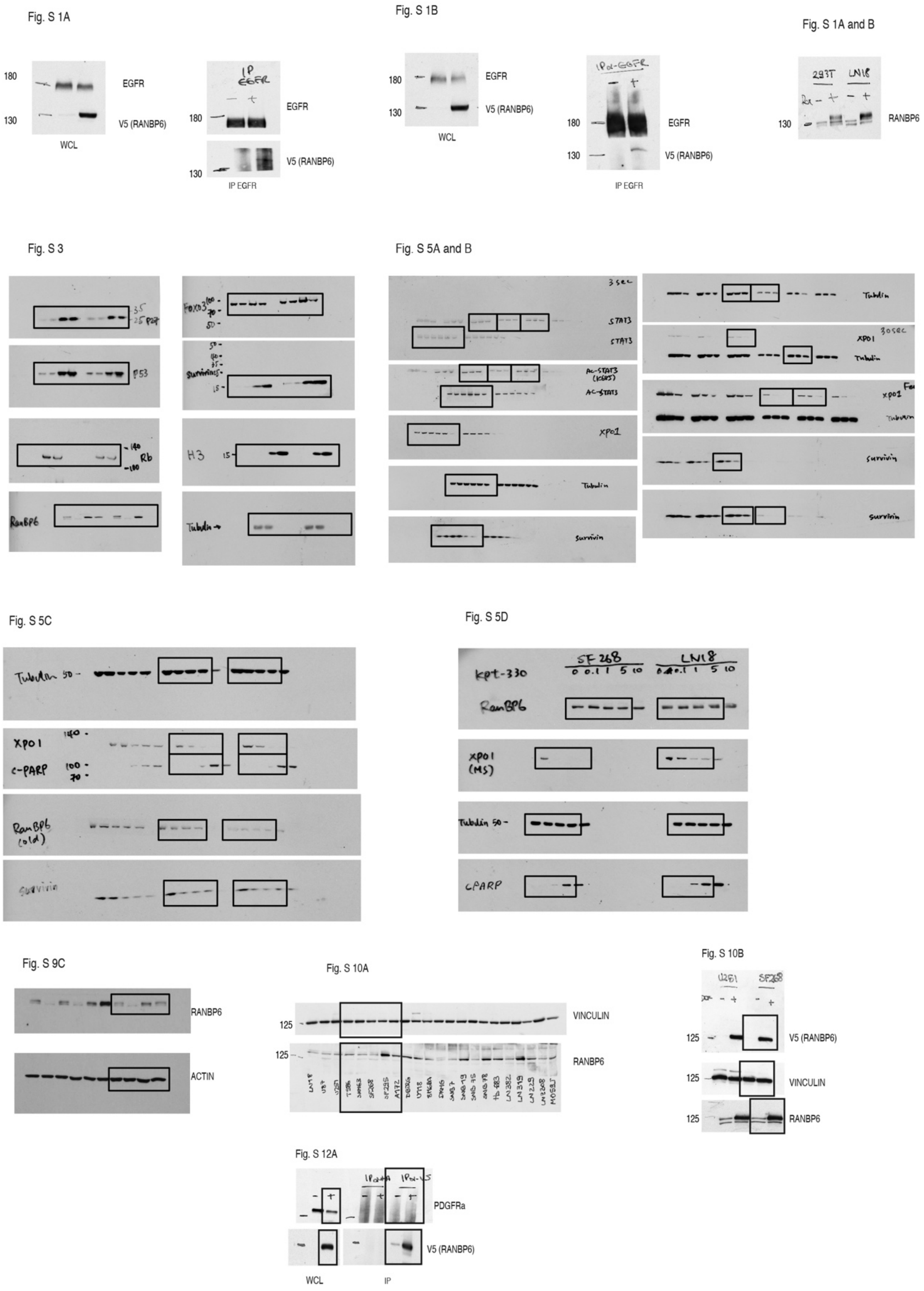

Supplementary Figure 15. Uncropped western blot images for Supplementary Figures

Supplementary Table 1: Predicted STAT3 binding site on EGFR promoter

Model_ID	Model_name	Score	Relative_score	Start	End	Strand	Predicted_site_sequence	Region
MA0144.2	STAT3	1.435	0.815	-1463	-1453	-1	CAAATGGGAAG	
MA0144.2	STAT3	0.529	0.804	-1355	-1345	1	ATTCAGGGCAT	
MA0144.2	STAT3	1.383	0.814	-1271	-1261	-1	CAACCTGGAAC	EGFR_1
MA0144.2	STAT3	0.543	0.804	-1166	-1156	-1	CATCCAGCAAA	EGFR_1
MA0144.2	STAT3	1.311	0.813	-1077	-1067	-1	CTGTCAAAAAT	
MA0144.2	STAT3	1.652	0.818	-857	-847	-1	ATGCCAGGGAA	
MA0144.2	STAT3	4.611	0.853	-766	-756	-1	GCTCTTGGAAT	
MA0144.2	STAT3	2.916	0.833	-751	-741	1	CTTTTGCGAAG	
MA0144.2	STAT3	1.288	0.813	-628	-618	1	GTGCTTGACAA	
MA0144.2	STAT3	1.746	0.819	-496	-486	1	CTGCAGGGGAG	
MA0144.2	STAT3	0.274	0.801	-375	-365	1	AAGAAGGGAAA	
MA0144.2	STAT3	10.95	0.93	-278	-268	1	GTGCTGGGAAC	EGFR_2
MA0144.2	STAT3	2.403	0.827	-264	-254	-1	TTTCCGAGAGG	EGFR_2
MA0144.2	STAT3	2.632	0.829	-263	-253	1	CTCTCGGAAAT	EGFR_2

Supplementary Table 2: Primers for cloning and mutagenesis

ID	Sequence (5' -> 3')
pGEX6p-RanBP6-Forward	ATTCCCGGGATGGCGGCAACCG
pGEX6p-RanBP6-Reverse	CGATGCGGCCGCTCAAGCAAAATTTAGCAACTCG
RanBP6-MIGR1-Forward	GAATTAGATCTACCATGGCGGCAACC
RanBP6-MIGR1-Reverse	GTTAACCTCGAGTCAAGCAAAATTTAGC
RanBP6-867 hairpin resistant cDNA sense	GAGAACTGTATCTCAGCAATAGGGAAAATTTTAAAGTTTAAACCTAACTGTGTAAATGTAGATG
RanBP6-867 hairpin resistant cDNA antisense	CATCTACATTTACACAGTTAGGTTTAAACTTTAAAATTTTCCCTATTGCTGAGATACAGTTCTC
pLenti6.3-RanBP6-V5 Forward	GGGGGACAAGTTTGTACAAAAAAGCAGGCTCCACCATGGCGGCAA
pLenti6.3-RanBP6-V5 Reverse	GGGGACCACTTTGTACAAGAAAGCTGGGTAAGCAAAATTTAG

Supplementary Table 3: Primers for silencing and CRISPR/Cas9 editing

Short-hairpin RNA target site sequences:

ID	Sequence (5' -> 3')
TRIPz sh Human RanBP6_1	TTAATTCCAAAACTCTGCT
TRIPz sh Human RanBP6_2	TAAACTTCAAAATCTTCCC
RCAS sh Mouse RanBP6	CCAGGTTGAAATGTCTCTTCA
RCAS sh Luciferase	TTAATCAGAGACTTCAGGCGGT
TRIPz sh Human STAT3	AAGTTTCTAAACAGCTCCA

Single-guided RanBP6 target site sequences:

ID	20nt sequence (5' -> 3')	PAM	Strand
1	ATGGCGGCAACCGCGTCTGC	AGG	+

Surveyor primers for PCR amplifying U6-sgRNA fragments:

ID	Sequences (5' -> 3')
RanBP6_S7F	CCAAAACCTAGCTCGCAGCC
RanBP6_S7R	GAAACTTCAGACCTTCCGGC
RanBP6_S8F	CACGGCTCTCACCTCTCTCC
RanBP6_S9R	GCCAGAAAACGTGAAGTGCAA

Primer for checking the indels of the PCR product:

ID	Sequences (5' -> 3')
Human U6 SeqF_Insert	ACTATCATATGCTTACCGTAAC

Supplementary Table 4: Primers for RT-qPCR and ChIP analysis

Primer for RT-qPCR analysis:

Gene symbol	Forward Primer 5’->3’	Reverse Primer5’->3’
Ranbp6 (mouse)	ATGATGACTGGGTAAATGCTGA	CCAAGTCCACAAGCCAGTCT
EGFR (human)	AGTCGGGCTCTGGAGGAAA	ACATCCTCTGGAGGCTGAGA
PDGFRA (human)	GAAGCTGTCAACCTGCATGA	CTTCCTTAGCACGGATCAGC
PDGFRB (human)	GTGAACGCAGTGCAGACTGT	AGGTGTAGGTCCCCGAGTCT
STAT3 (human)	CAGGAGGGAGCTGTATCAGG	AGGACTTGGGCACAGAAGC
PTGS2 (human)	AGGGATTTTGGAACGTTGTG	GAGAAGGCTTCCCAGCTTTT
MAFF (human)	AGCGGAGGGGAGACTGAC	CACAGACATGTTTGCAGAAGG
EFNB2 (human)	TCTTTGGAGGGCCTGGAT	GATCCAGCAGAACTTGCATCT
ITIF (human)	AGAACGGCTGCCTAATTTACAG	GCTCCAGACTATCCTTGACCTG
CPS1 (human)	CAAGTTTTGCAGTGGAATCG	ACTGGGTAGCCAATGGTGTC
HPRT (human)	GGCCAGACTTTGTTGGATTTG	TGCGCTCATCTTAGGCTTTGT

Primer for ChIP analysis:

ID	Forward Primer 5’->3’	Reverse Primer5’->3’
EGFR_ STAT3_1	CAACGCACAGTGGCTGTACT	CCCTTTGCTGTCTCTGAAGG
EGFR_ STAT3_2	TTGGCTCGACCTGGACATAG	GAGGGAGGAGAACCAGCAG
PTGS2_ STAT3_1	AACCTTACTCGCCCCAGTCT	CCGCCAGATGTCTTTTCTTC
HPRT	CGGTAGGTTTGGGAATCA	CAGTTTGCAGGCTCACTA
